# Structural insights into ubiquitin phosphorylation by PINK1

**DOI:** 10.1038/s41598-018-28656-8

**Published:** 2018-07-10

**Authors:** Kei Okatsu, Yusuke Sato, Koji Yamano, Noriyuki Matsuda, Lumi Negishi, Akiko Takahashi, Atsushi Yamagata, Sakurako Goto-Ito, Masaki Mishima, Yutaka Ito, Toshihiko Oka, Keiji Tanaka, Shuya Fukai

**Affiliations:** 1 Institute for Quantitative Biosciences, The University of Tokyo, Tokyo, 113-0032 Japan; 2 Synchrotron Radiation Research Organization, The University of Tokyo, Tokyo, 113-0032 Japan; 3 Department of Computational Biology and Medical Sciences, Graduate School of Frontier Sciences, The University of Tokyo, Chiba, 277-8561 Japan; 4 Tokyo Metropolitan Institute of Medical Science, Tokyo, 156-8506 Japan; 5 PRESTO, Japan Science and Technology Agency, Saitama, 332-0012 Japan; 6 Graduate School of Science & Engineering, Tokyo Metropolitan University, Tokyo, 192-0397 Japan; 7 Department of Life Science, Rikkyo University, Tokyo, 171-8501 Japan

## Abstract

Mutations of PTEN-induced putative kinase 1 (PINK1) and the E3 ubiquitin (Ub) ligase parkin can cause familial parkinsonism. These two proteins are essential for ubiquitylation of damaged mitochondria and subsequent degradation. PINK1 phosphorylates Ser65 of Ub and the Ub-like (UBL) domain of parkin to allosterically relieve the autoinhibition of parkin. To understand the structural mechanism of the Ub/UBL-specific phosphorylation by PINK1, we determined the crystal structure of *Tribolium castaneum* PINK1 kinase domain (*Tc*PINK1) in complex with a nonhydrolyzable ATP analogue at 2.5 Å resolution. *Tc*PINK1 consists of the N- and C-terminal lobes with the PINK1-specific extension. The ATP analogue is bound in the cleft between the N- and C-terminal lobes. The adenine ring of the ATP analogue is bound to a hydrophobic pocket, whereas the triphosphate group of the ATP analogue and two coordinated Mg ions interact with the catalytic hydrophilic residues. Comparison with protein kinases A and C (PKA and PKC, respectively) unveils a putative Ub/UBL-binding groove, which is wider than the peptide-binding groove of PKA or PKC to accommodate the globular head of Ub or UBL. Further crosslinking analyses suggested a PINK1-interacting surface of Ub. Structure-guided mutational analyses support the findings from the present structural analysis of PINK1.

## Introduction

PTEN-induced putative kinase 1 (PINK1) is a mitochondrial serine/threonine kinase that accumulates and is activated on mitochondria, following a decrease in mitochondrial membrane potential^1–3^. The activated PINK1 phosphorylates ubiquitin (Ub) and the Ub-like domain (UBL) of the E3 Ub ligase parkin at Ser65^4–8^. The phosphorylation of Ub and the parkin UBL facilitates the transition from the autoinhibition state to the catalytically active state in parkin to drive degradation of depolarized mitochondria. Structural studies on parkin have elucidated the mechanism of its autoinhibition and allosteric activation^5,9–15^. In contrast, the structural mechanism of the Ub phosphorylation by PINK1 was unclear when we started this study. It has been reported that *Tribolium castaneum* PINK1 kinase domain (hereafter referred to as *Tc*PINK1) expressed in *Escherichia coli* has strong kinase activity to Ub^5,7,16^. We thus selected *Tc*PINK1 as a target for crystallography. In this study, we determined the crystal structure of *Tc*PINK1 in complex with a nonhydrolyzable ATP analogue (adenosine 5′-(β-γ-imido)triphosphate; AMP-PNP). The mechanism for Ub phosphorylation by PINK1 was further investigated by structure-guided mutational analyses at the molecular and cellular levels. The findings from this study were compared with those from the recently reported structures of apo-*Tc*PINK1 and the Ub (T66V N67L; Ub^TVNL^)-bound *Pediculus humanus corporis* PINK1 kinase domain (hereafter referred to as *Ph*PINK1)^17,18^.

## Results and Discussion

### Identification of autophosphorylation sites of *Tc*PINK1

The conformation and activity of protein kinases are regulated by (auto)phosphorylation in general^19^. Phosphorylation in the activation loop can change its conformation from the inactive state to the active state. Alternatively, phosphorylation on the distal surface of the catalytic site can be allosterically coupled with the kinase activity^20^. It has been reported that *Homo sapiens* PINK1 (*Hs*PINK1) is phosphorylated at Ser228 and Ser402, whereas *Tc*PINK1 is phosphorylated at Ser205^1,16^. We first tested the autophosphorylation levels of the wild-type and kinase-dead (KD; D359A) *Tc*PINK1 proteins, using phosphate affinity SDS-PAGE (Phos-tag SDS-PAGE). Phos-tag is a small molecule that binds to acrylamide with a covalent bond and coordinates manganese ions. Phosphorylated proteins are migrated slower than non-phosphorylated proteins in Phos-tag-containing gel and thereby separated from them^21^. Wild-type *Tc*PINK1 was stained as smear bands in Phos-tag gel, whereas the KD *Tc*PINK1 was detected as a single sharp band (Supplementary Fig. 1A), suggesting that wild-type *Tc*PINK1 was hyperphosphorylated in *E*. *coli*. Heterogeneous phosphorylation of wild-type *Tc*PINK1 inhibited its crystallization, but the KD *Tc*PINK1 tended to aggregate.

**Figure 1:**
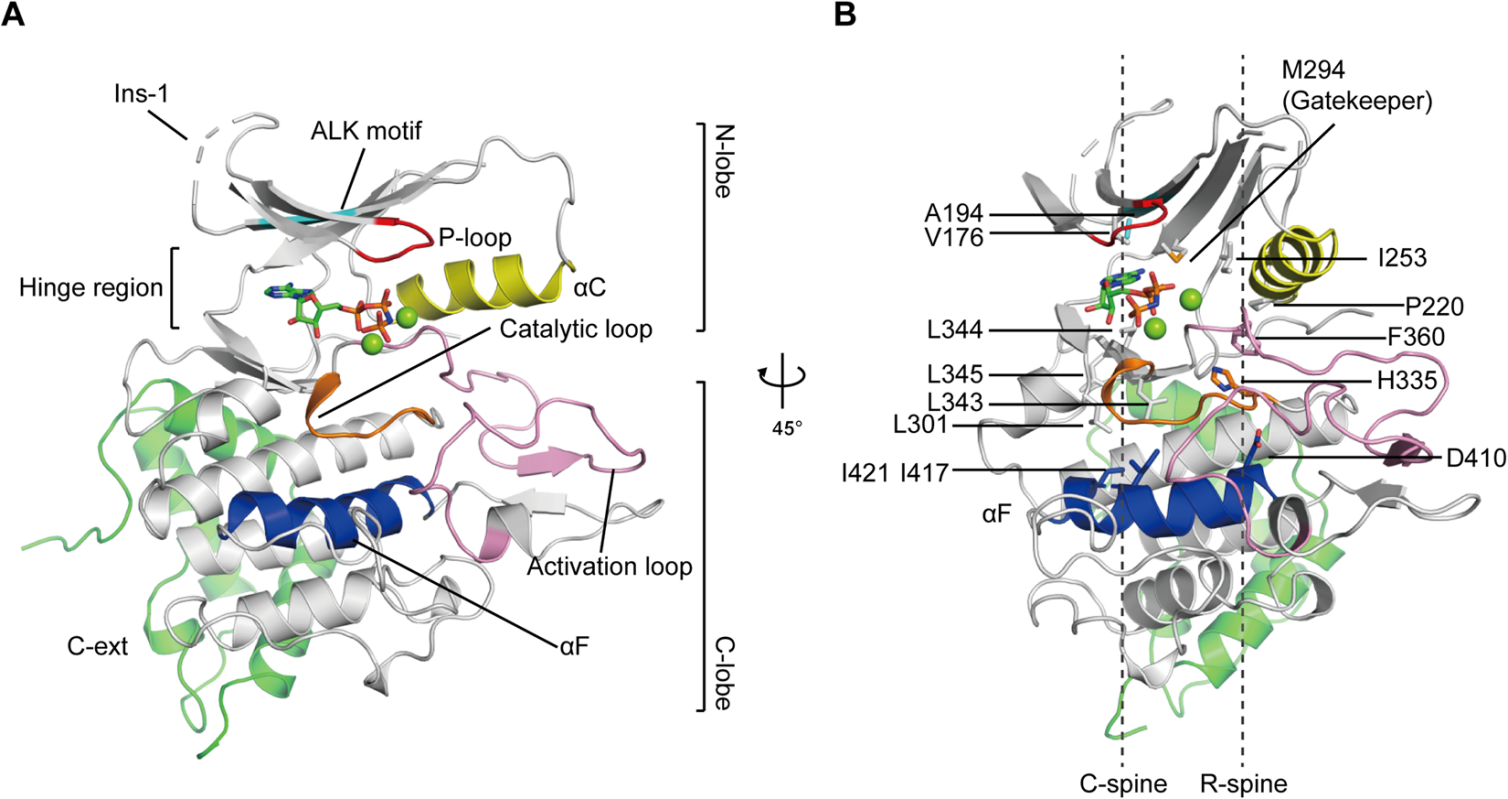
Architecture of *Tc*PINK1. (**A**) Crystal structure of *Tc*PINK1^DDEE^ in complex with an ATP analogue. The bound ATP analogue is shown as sticks. The two coordinated Mg ions are shown as green spheres. The colouring scheme is as follows: P-loop, red; ALK motif, cyan; αC, yellow; catalytic loop, orange; activation loop, pink; αF, blue; C-ext, green. (**B**) C- and R-spines in *Tc*PINK1^DDEE^. The C- and R-spines are indicated by dashed lines. The gatekeeper residue and residues involved in the C- and R-spines are shown as sticks. The colouring scheme is the same as that in (**A**).

To obtain stable PINK1 proteins with high purity for crystallisation, we aimed to design *Tc*PINK1 mutants that could be phosphorylated homogeneously. We searched for the autophosphorylation sites of *Tc*PINK1 by Ala replacement of the potential autophosphorylation serine/threonine residues. First, Ser205 and Ser377 of *Tc*PINK1, which correspond to the previously reported phosphorylation sites of *Hs*PINK1 (Ser228 and Ser402)^1,16^, were mutated. The autophosphorylation level of *Tc*PINK1 was decreased by the S205A mutation and further by the S205A S377A double mutation (Supplementary Fig. 1B, left). We next mutated four serine/threonine residues in the activation loop of *Tc*PINK1 (*i*.*e*., Thr368, Ser372, Thr376 and Thr386). The T386A mutation decreased the autophosphorylation level, whereas the T368A, S372A or T376A mutation did not, suggesting that Thr386 is another autophosphorylation site (Supplementary Fig. 1B, right). We then prepared the phosphomimetic S205D S377D T386E triple mutant of *Tc*PINK1 and analysed it by Phos-tag SDS-PAGE (Supplementary Fig. 1C). This phosphomimetic mutant was detected as two major sharp bands and weak smear bands in Phos-tag gel, suggesting the presence of additional phosphorylation sites. To identify them, we performed LC-MS/MS analysis after separating the phosphorylated triple mutant by Phos-tag SDS-PAGE and detected peptides phosphorylated at Thr530 (Supplementary Fig. 1C). The phosphomimetic S205D S377D T386E T530E quadruple mutant of *Tc*PINK1 (hereafter referred to as *Tc*PINK1^DDEE^) migrated as a single major band and weak smear bands in Phos-tag gel (Supplementary Fig. 1D). The weak smear bands, which may correspond to *Tc*PINK1^DDEE^ phosphorylated at minor phosphorylation sites, mostly disappeared after treatment with lambda protein phosphatase (Supplementary Fig. 1E). The phosphatase-treated *Tc*PINK1^DDEE^ yielded high-quality crystals suitable for crystallography. *Tc*PINK1^DDEE^ has residual Ub phosphorylation activity (Supplementary Fig. 1F). Finally, we successfully determined the crystal structure of *Tc*PINK1^DDEE^ in complex with AMP-PNP (Fig. 1A and Supplementary Table 1).

### Overall structure of *Tc*PINK1^DDEE^ in complex with AMP-PNP

*Tc*PINK1^DDEE^ consists of the N- and C-terminal lobes (residues 153–295 and 301–485; N- and C-lobes, respectively) with the PINK1-specific extension (C-ext; residues 486–570) (Fig. 1A). The ATP analogue is bound in the cleft between the N- and C-lobes. The N-lobe contains a five-stranded antiparallel β-sheet (β1–β5), an α-helix (known as ‘αC-helix’; αC) (Supplementary Figs 2 and 3) and three PINK1-specific insertions (Supplementary Fig. 2): the first insertion (Ins-1) forms a loop containing acidic amino-acid residues, although the electron density of residues 184–190 and the Lys180 and Tyr190 side chains is invisible, probably owing to their structural disorder. The second and third insertions (Ins-2 and Ins-3, respectively) are disordered in the present *Tc*PINK1^DDEE^ structure (Fig. 1A and Supplementary Fig. 2). Ins-1 is not conserved in *Hs*PINK1. The phosphate-binding loop (P-loop), which contains conserved glycine residues, connects β1 and β2. Lys196 on β3 in the ALK motif (^194^Ala-Leu-Lys^196^) contacts the invariable Glu217 in αC. The C-lobe is composed of αD–αI with the catalytic and activation loops (Fig. 1A and Supplementary Fig. 3). The catalytic loop has the conserved HRD motif (^335^His-Arg-Asp^337^). The region between the DFG (^359^Asp-Phe-Gly^361^) and APE motifs (^390^Ala-Pro-Glu^392^) was defined as the activation loop. The C-ext consists of three α-helices (αJ–αL) (Supplementary Figs 2 and 3), which contact the distal surface of the kinase domain apart from the active site through extensive hydrophobic interactions (Supplementary Fig. 4A) and the Arg549-mediated hydrogen bonds with Glu323 and Asn478 (Supplementary Fig. 4B). The present AMP-PNP-bound *Tc*PINK1^DDEE^ structure is similar to the apo-*Tc*PINK1 (E527A K528A S205E Δ261–270) structure (Cα rmsd of 1.1 Å)^17^, except that the N-lobe is shifted by approximately 5 Å (Supplementary Fig. 5A). Structural comparison with the Ub^TVNL^-bound *Ph*PINK1 showed the difference in Ins-3, which is ordered and interacts with Ub in the Ub^TVNL^-bound structure^18^. Concomitantly, αC is rearranged upon binding to Ub (Supplementary Fig. 5B). The phosphorylation of Ser205 contributes to the conformational change in Ins-3, which is stabilized in the Ub-bound state^18,22^.

### Conformational features of AMP-PNP-bound *Tc*PINK1^DDEE^

The active kinase conformation is canonically defined by the so-called ‘DFG Asp-in conformation’ and ‘Glu-Lys contact’^23^. In the present AMP-PNP-bound *Tc*PINK^DDEE^ structure, Asp359 is located inside the protein and coordinates the two catalytic magnesium ions with the triphosphate group of the bound AMP-PNP. Glu217 of *Tc*PINK1^DDEE^ interacts with Lys196 and also coordinates one of the two catalytic magnesium ions. The details of these interactions are described in the subsection ‘ATP recognition and Mg^2+^ coordination’. The active conformation is also characterized by the assembly of ‘hydrophobic spines’ specified by local spatial pattern alignment of kinases of known structure^24^. Such spines are classified into the catalytic and regulatory spines (C- and R-spines, respectively). A ‘gatekeeper’ residue is located between the R- and C-spines. In *Tc*PINK1^DDEE^, the C-spine consists of Val176, Ala194, Leu301, Leu343, Leu344, Leu345, Ile417 and Ile421, whereas the R-spine consists of Pro220, Ile253, His335 and Phe360 (Fig. 1B). The gatekeeper residue is Met294. Ile253 and Pro220 in the R-spine are located in β4 and αC, respectively. His335 and Phe360 are involved in the HRD and DFG motifs, respectively. Asp410 in αF interacts with His335 in the R-spine. Val176 in β2 and Ala194 in β3 in the C-spine and Leu344 in β7 sandwich the adenine ring of the bound ATP analogue. Leu343 and Leu345 flanked by Leu344 interact with Leu301 in αD and Ile417 in αF. Ile421 in αF also stabilizes the C-spine by the interaction with Leu301. Therefore, αF anchors the C- and R-spines. The present *Tc*PINK1^DDEE^ structure defines the key residues composing the spines. The linear spines in this AMP-PNP-bound *Tc*PINK1^DDEE^ structure represent the active kinase conformation^24^ and are similar to those in the apo-*Tc*PINK1 and Ub^TVNL^-bound *Ph*PINK1 structures^17,18^.

### Pathogenic mutations

Mutations of *Hs*PINK1 can cause autosomal recessive parkinsonism^25^. Two recent structural studies on PINK1 comprehensively explained the influence of pathogenic mutation in PINK1^17,18^. We here mention that the *Tc*PINK1 residues corresponding to eight pathogenic mutation sites within the kinase domain of *Hs*PINK1 are located on or in close proximity to αF^26,27^ (Supplementary Table 2). These residues are mostly hydrophobic and form hydrophobic cores around αF, which anchors the C- and R-spines, in *Tc*PINK1^DDEE^ (Supplementary Fig. 6A and B). The changes in the size and/or hydrophobicity of the side chains by the mutations may destabilize αF and thereby disturb the R- and C-spines, which are closely associated with the kinase activity. αF also interacts with the activation loop to stabilize its conformation for the kinase activity. The proline residue (Pro391 or Pro416 in *Tc*PINK1 or *Hs*PINK1, respectively) in the APE motif is a key residue for this interaction (Supplementary Fig. 6C) and its mutation to Arg has been reported to be pathogenic^26^. The E417G mutation of *Hs*PINK1 is another pathogenic mutation in the APE motif. The corresponding Glu392 of *Tc*PINK1 forms hydrogen bonds with Arg470 (corresponding to Arg497 of *Hs*PINK1) so as to adjust the position of Pro391, which interacts with Trp412 on αF (Supplementary Fig. 6D). Consistently, the E417G mutation of *Hs*PINK1 is defective in the kinase activity^1,16^.

### ATP recognition and Mg^2+^ coordination

**Figure 2:**
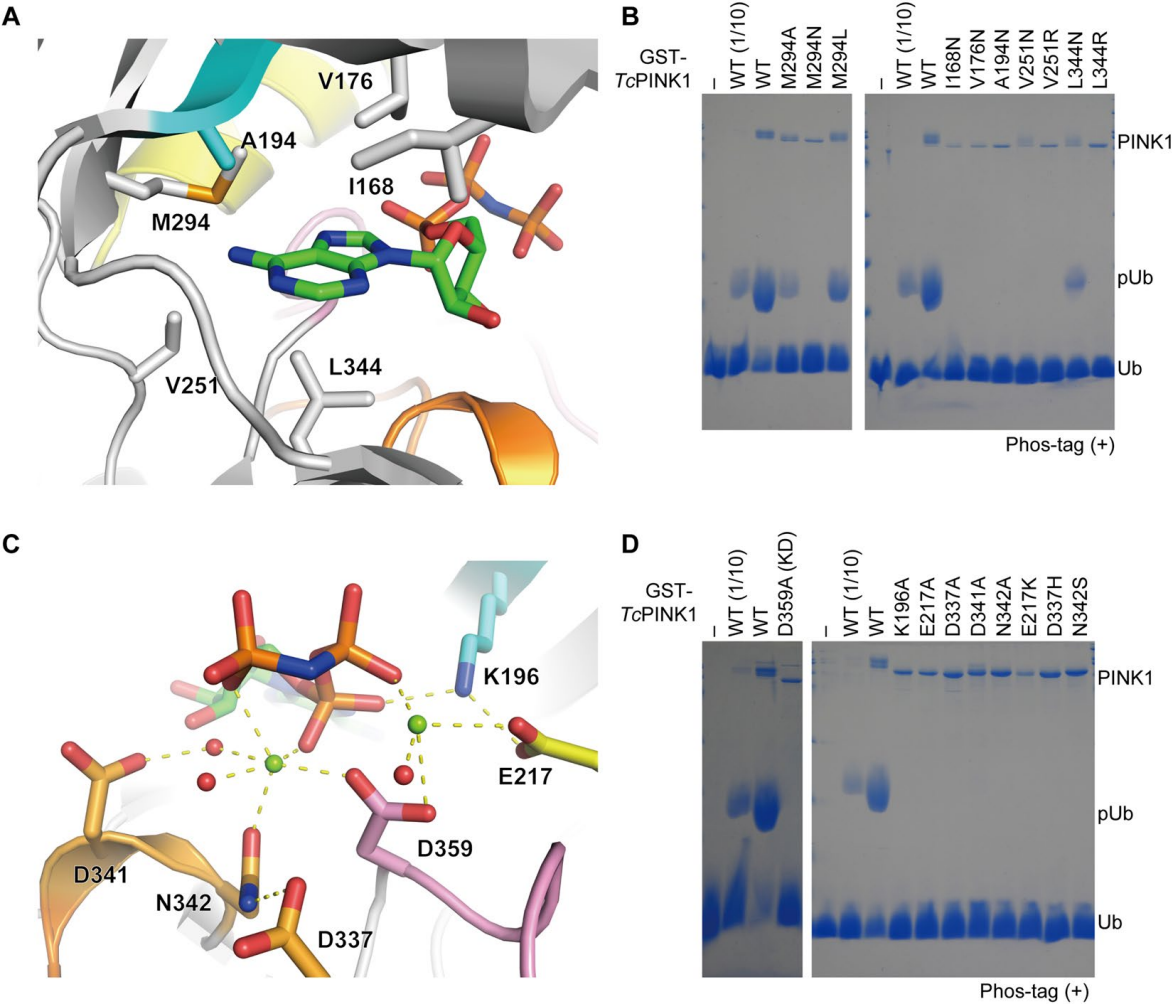
Catalytic site of *Tc*PINK1. (**A**) Recognition of the adenine ring of the bound ATP analogue. The adenine ring-interacting residues and bound ATP analogue are shown as sticks. The colouring scheme is the same as that in Fig. 1A. (**B**) *In vitro* kinase assays of *Tc*PINK1 mutants that were designed to compromise the interaction with the adenine ring of the ATP analogue. The Ub phosphorylation (pUb) by wild-type or mutant GST-*Tc*PINK1 was analysed by Phos-tag SDS-PAGE with Coomassie brilliant blue staining. (**C**) Mg^2+^-mediated interactions with the triphosphate group of the bound ATP analogue. The Mg^2+^-coordinating residues and bound ATP analogue are shown as sticks. The coordinated Mg ions and water molecules are shown as green and red spheres, respectively. The interactions are indicated by dashed lines. The colouring scheme is the same as that in Fig. 1A. (**D**) *In vitro* kinase assays of *Tc*PINK1 mutants that were designed to compromise the coordination of the catalytic Mg ions. The Ub phosphorylation (pUb) by wild-type or mutant GST-*Tc*PINK1 was analysed by Phos-tag SDS-PAGE with Coomassie brilliant blue staining. KD; kinase dead. 1/10; one-tenth dose of GST-*Tc*PINK1.

Kinases catalyse the transfer of the γ-phosphate of ATP to their substrates. The adenine ring of the AMP-PNP molecule bound to *Tc*PINK1^DDEE^ is surrounded by hydrophobic residues including Met294, the gatekeeper residue of *Tc*PINK1^DDEE^ (Fig. 2A). To assess the functional importance of the adenine ring-interacting hydrophobic residues *in vitro*, we replaced Ile168, Val176, Ala194, Val251, Met294 and Leu344 of *Tc*PINK1 by alanine, hydrophilic or pathogenic residues and tested their effects on autophosphorylation and Ub phosphorylation. The M294A and M294L mutations decreased the Ub phosphorylation as equivalent to one-tenth dose of wild-type PINK1, whereas the M294N mutation completely abolished it (Fig. 2B, left and Supplementary Fig. 7). The other mutations also decreased or abolished the Ub phosphorylation (Fig. 2B, right and Supplementary Fig. 7). The autophosphorylation was decreased by all the mutations examined, although the V251N and L344N mutations were less effective than the other mutations (Supplementary Fig. 8). These two mutations may affect both ATP hydrolysis and Ub binding. The impact on Ub binding is likely to be indirect, since Val251 and Leu344 are located far from the Ub-binding site, which is mentioned in the next subsection. These results showed that the hydrophobic pocket of PINK1 for the adenine ring recognition is critically important for the Ub phosphorylation and autophosphorylation.

The triphosphate group of the bound ATP analogue and surrounding hydrophilic residues of *Tc*PINK1^DDEE^ coordinate two Mg ions (Fig. 2C). One Mg^2+^ is coordinated by the α- and γ-phosphate groups of the bound ATP analogue, Asn342 and Asp359 of *Tc*PINK1, and two water molecules. One of these two water molecules and Asn342 form hydrogen bonds with Asp341 and Asp337, respectively, in the catalytic loop. The mutations of Glu217, Asp337 and Asn342 in *Tc*PINK1 decreased the Ub phosphorylation by *Tc*PINK1 (Fig. 2D and Supplementary Fig. 7). Glu217, Asp337 and Asn342 of *Tc*PINK1 correspond to Glu240, Asp362 and Asn367 of *Hs*PINK1, respectively, which are associated with pathogenesis^26^, and equivalent mutation in *Tc*PINK1 again decreased the Ub phosphorylation (Fig. 2D and Supplementary Fig. 7). The other Mg^2+^ is coordinated by the β-phosphate group of the bound ATP analogue, Glu217 and Asp359 of *Tc*PINK1, and one water molecule (Fig. 2C). Glu217 in αC hydrogen bonds with Lys196 in the ALK motif, as generally observed in the active kinase conformation, although the electron density of the Lys196 side chain is weak. Asp359 in the DFG motif is involved in the coordination of both Mg ions. Correspondingly, the D359A mutation completely abolished the kinase activity of *Tc*PINK1 *in vitro* (Fig. 2D and Supplementary Fig. 7)^16^.

### Insights into the interaction between PINK1 and Ub

**Figure 3:**
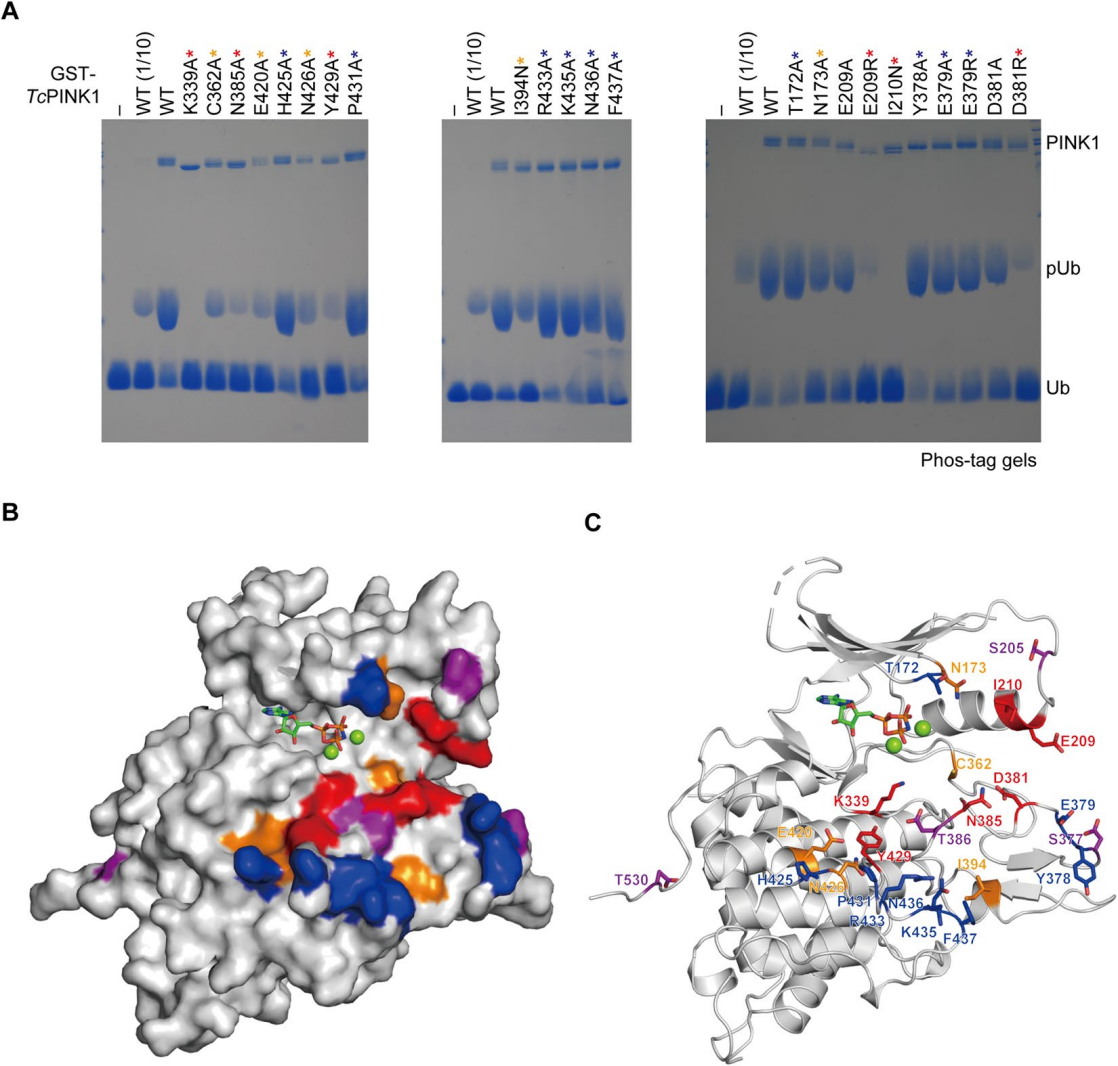
Putative Ub-binding groove of *Tc*PINK1. (**A**) *In vitro* kinase assays of *Tc*PINK1 mutants that were expected to compromise the interaction with Ub. The Ub phosphorylation (pUb) by wild-type or mutant GST-*Tc*PINK1 was analysed by Phos-tag SDS-PAGE with Coomassie brilliant blue staining. The colour-coded asterisks represent the magnitude of the effect of each mutation on the Ub phosphorylation: red, drastic (less than one-tenth dose of wild type); orange, mild (less than wild type); blue, little or no (nearly equal to wild type). (**B**,**C**) Mapping of potential Ub-interacting residues on the molecular surface (**B**) and cartoon model (**C**) of *Tc*PINK1^DDEE^. The potential Ub-interacting residues are shown as sticks in (**C**). The colour-coding is based on the magnitude of the effect of each mutation on Ub phosphorylation, except that phosphomimetic sites are coloured in purple: red, drastic (less than one-tenth dose of wild type); orange, mild (less than wild type); blue, little or no (nearly equal to wild type).

The complex structures of protein kinase A (PKA; also known as c-AMP protein kinase) and protein kinase C (PKC) with substrate peptides have revealed the key residues for their substrate specificities, which are distributed in the P-loop, αC, αD, αF, αG and activation loop^28–30^. These structural elements form an approximately 15-Å-wide groove to accommodate the substrate peptide (Supplementary Fig. 9A and B). On the other hand, extracellular signal-regulated kinase 2 (ERK2) and LIM domain kinase 1 (LIMK1) and have approximately 22- and 24-Å-wide grooves to accommodate globular proteins, ribosomal S6 kinase 1 (RSK1) and cofilin/ADF, respectively (Supplementary Fig. 9C and D)^31,32^. *Tc*PINK1^DDEE^ similarly forms a 23-Å-wide groove (Supplementary Fig. 9E), consistent with the fact that PINK1 recognizes Ub as a substrate. The potential PINK1-binding surface of Ub is positively charged (Supplementary Fig. 10A), corresponding to the negatively charged feature of the potential substrate-binding groove of *Tc*PINK1 (Supplementary Fig. 10B). In contrast, the groove of ERK2 is positively charged in contrast to that of PINK1 (Supplementary Fig. 10C). The electrostatic feature of the potential substrate-binding groove of PINK1 may be important for the specificity for Ub. The functional relevance of this groove to Ub phosphorylation was demonstrated by introducing the mutations of residues on the groove and testing their effects on the kinase activities for Ub: the E209R, I210N, D381R, N385A and Y429A mutations of *Tc*PINK1 drastically reduced the activity (less than one-tenth dose of wild type), whereas the C362A, E420A and N426A mutations mildly decreased it (less than wild type but more than one-tenth dose of wild type) (Fig. 3 and Supplementary Fig. 11). The other examined mutations showed the kinase activity equivalent to that of wild type. The drastic effects of the D381R, N385A and Y429A mutations of *Tc*PINK1 on the Ub phosphorylation could be interpreted on the basis of the Ub^TVNL^-bound *Ph*PINK1 structure: Asp379 of *Ph*PINK1 (Asp381 in *Tc*PINK1) contacts the side chain of His68 in Ub, whereas Asn383 in *Ph*PINK1 (Asn385 in *Tc*PINK1), which is stabilised by the interaction with Tyr427 of *Ph*PINK1 (Tyr429 in *Tc*PINK1), contacts the main-chain NH group of Val66 in Ub (Supplementary Fig. 12). Ala206 and Ile207 of *Ph*PINK1 (Glu209 and Ile210 in *Tc*PINK1, respectively) are positioned in αC, which is spatially rearranged upon binding to Ub, and thereby located far from the bound Ub. Mutations of these two residues might inhibit this rearrangement of αC, although we cannot exclude the possibility that αC of *Tc*PINK1 is not rearranged upon binding to Ub and can be located close to Ub in the complex.

We next asked which regions of Ub or the parkin UBL interact with PINK1. NMR ^15^N-^1^H HSQC analysis of the ^15^N-labeled Ub was carried out in the presence of *Tc*PINK1 and AMP-PNP. In this condition, the result should reflect the binding to *Tc*PINK1 but not the phosphorylation at Ser65, which has been extensively studied by NMR^33–35^. The intensity ratios (*I*_per_/*I*_ref_) of Ub at Lys6, Ile44 and Ala46 were clearly decreased (Supplementary Fig. 13). To further examine the interaction mode between Ub and PINK1, the photoreactive amino acid *p*-benzoyl-phenylalanine (BPA) was comprehensively introduced to Ub in a site-specific manner and reacted to *Tc*PINK1. The selected mutation sites are located on the surface of Ub and unlikely to disturb the core structure of Ub. The crosslinked products were then analysed by SDS-PAGE with Coomassie brilliant blue staining (Supplementary Fig. 14A and B). The substitution of BPA for Gly10, Gln49 or Arg72 generated the clear band of the crosslinked product, whereas that for Glu2, Lys6, Lys11, Glu24, Asp32, Glu34, Arg42, Ile44, Thr66 or His68 generated the weak band. Mapping of these crosslinked sites on the Ub structure suggested that the Ile44-centered hydrophobic patch of Ub is the primary interface for PINK1 (Supplementary Fig. 14C). Indeed, the major crosslinked sites of Ub (*i*.*e*., Gly10, Gln49 and Arg72) are located close to PINK1 in the structure of the Ub^TVNL^–*Ph*PINK1 complex (Supplementary Fig. 15)^18^.

### Structure-guided mutational analysis on PINK1-mediated parkin activation in cells

**Figure 4:**
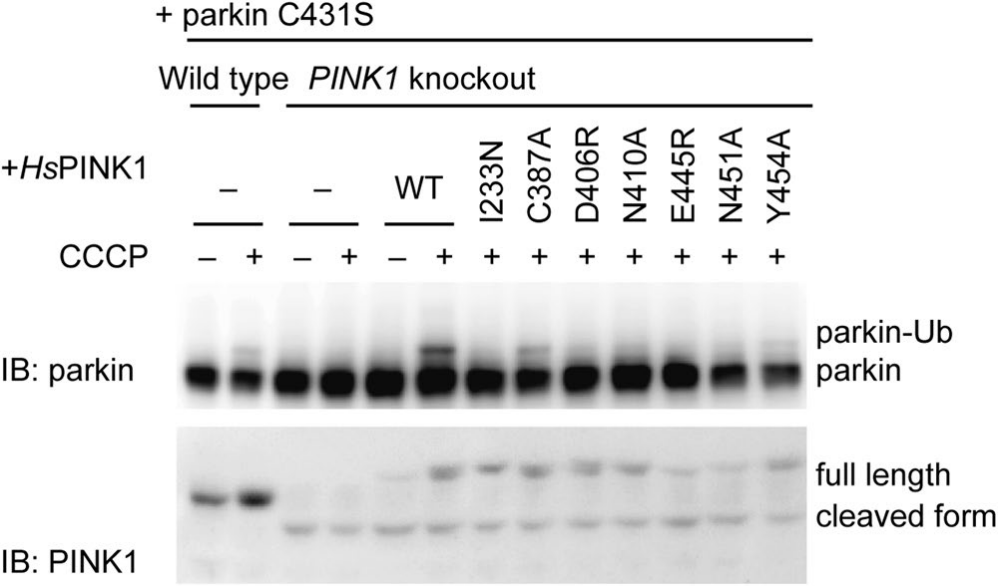
Inhibition of parkin activation by structure-guided mutations of *Hs*PINK1. Formation of the Parkin-Ub oxyester intermediate in the *PINK1*-knockout HeLa cells expressing HA-parkin (C431S) and wild-type or mutant *Hs*PINK1-3xHA after CCCP treatment was examined by immunoblotting analysis using anti-parkin and anti-PINK1 antibodies.

PINK1 acts upstream of parkin to promote ubiquitylation of depolarized mitochondria^2,3,36^. To investigate the impacts of the catalytically deficient mutants of PINK1 at the cellular level, we examined the activation of parkin after mitochondrial depolarization in *PINK1*-knockout cells heterologously coexpressing wild-type or mutant *Hs*PINK1 and the parkin C431S mutant. The key residues involved in the kinase activity of *Tc*PINK1 are shown in Fig. 3. Among them, we mutated the residues conserved between *Hs*PINK1 and *Tc*PINK1 (Ile233, Cys387, Asp406, Asn410, Glu445, Asn451 and Tyr454 of *Hs*PINK1, which correspond to Ile210, Cys362, Asp381, Asn385, Glu420, Asn426 and Tyr429 of *Tc*PINK1, respectively). Parkin belongs to the RING-between-RING (RBR) E3 family, which transfers Ub from E2 to a substrate *via* the thioester intermediate between Ub and the catalytic Cys^37^. To detect the parkin activation and parkin-Ub intermediate formation, we mutated the catalytic Cys431 to Ser to covert unstable thioester linkage to stable oxyester linkage^6,38^. Wild-type and *PINK1*-knockout HeLa cells coexpressing the aforementioned PINK1 mutants and parkin C431S mutant were treated with the uncoupler carbonyl cyanide *m*-chlorophenyl hydrazone (CCCP) to depolarise mitochondrial membrane potential. The expression of wild-type *Hs*PINK1 recovered the oxyester formation of the parkin C431S mutant in *PINK1*-knockout cells, whereas the expression of the catalytically deficient mutant (*i*.*e*., I233N, D406R, N410A, E445R or N451A) did not. The oxyester formation in the *Hs*PINK1 (C387A)- or *Hs*PINK1 (Y454A)-expressing cells was less than that in the wild-type *Hs*PINK1-expressing cells (Fig. 4). The PINK1 residues that are important for Ub phosphorylation *in vitro* are consistently important for the phosphorylation-mediated activation of parkin in the cells. Additionally, the expression levels of the E445R and N451A mutants of *Hs*PINK1 appear less than those of wild type and the other mutants, implying that these two mutations might impact the stability of the full-length *Hs*PINK1.

In this study, we provide insights into the recognition of ATP and Ub by PINK1. Similarly to the structures of apo-*Tc*PINK1 and the Ub^TVNL^-bound *Ph*PINK1^17,18^, the AMP-PNP-bound *Tc*PINK1^DDEE^ structure exhibits canonical architecture of protein kinases, where the C-lobe is supported by the PINK1-unique C-terminal extension through extensive hydrophobic interactions and some hydrophilic interactions. Our crosslinking analysis is consistent with the Ub-binding region revealed by the structure of the Ub^TVNL^–*Ph*PINK1 complex^18^. Recently, an ATP analogue, kinetin riboside triphosphate (KTP), has received increasing attention as an *Hs*PINK1 activator^39^. We superposed a kinetin riboside monophosphate (KMP; derived from PDB 4KXN) onto the bound AMP-PNP in the AMP-PNP–*Tc*PINK1^DDEE^ complex so as to fit the adenine ring, ribose and α-phosphate moieties (Supplementary Fig. 16). In this superposition, the *N*6 furfuryl modification of the adenine ring causes a steric hindrance to the main chain of the hinge region connecting the N- and C-lobes, which might affect their spatial arrangement and kinase activity. Nevertheless, the present AMP-PNP-bound PINK1 structure might be helpful for the future design of another ATP analogue capable of regulating the kinase activity of PINK1.

## Materials and Methods

### Plasmids

The gene encoding the kinase domain of *Tc*PINK1 (residues 153–570) was PCR-amplified from *Tc*PINK1(128–570)^8^. To produce the N-terminal GST-fused *Tc*PINK1, the amplified gene was cloned into the pGEX-6P1 expression vector using *Bam*HI and *Xho*I. To produce the N-terminal His_6_-SUMO-fused *Tc*PINK1, the amplified gene digested with *Bam*HI and *Xho*I was cloned into the pCold-SUMO expression vector^40^ digested with *Bam*HI and *Sal*I. The synthesised DNA fragments of the lambda protein phosphatase gene were purchased from Life Technologies. The gene encoding lambda protein phosphatase was amplified by PCR and cloned into the pColdI vector using *Nde*I and *Xho*I to produce the N-terminal His_6_-tagged protein. pEVOL-pBpF (a gift from Peter Schultz; Addgene plasmid #31190)^41^ was used for the incorporation of BPA to Ub. For the cell-based assay, the genes encoding wild-type *Hs*PINK1 and human parkin C431S mutant were cloned into the pCMV(d3)TNT and pcDNA3.1 vectors, respectively. The *Hs*PINK1 expression vector, pCMV(d3)TNT, was generated by deletion of the upstream 676 bp of the CMV promoter in pCMVTNT plasmid (Promega). The fragment of the CMV promoter was amplified by PCR using 5′-CGTTGAGATCTATGGGCGGTAGGCGTGTACGGTG-3′ and 5′-AGTGCCTCACGACCAACTTCTG-3′ primers and digested with *Bgl*II and *Hind*III. The resultant 83-bp fragment was inserted into pCMVTNT instead of the CMV promoter. Mutations were generated by PCR-based mutagenesis.

### Sample preparation for crystallisation

*E*. *coli* strain Rosetta (DE3) (Invitrogen) cells were transformed with the pGEX-6P1 vector containing the gene encoding *Tc*PINK1^DDEE^ (residues 153–570 with the phosphomimetic S205D S377D T386E T530E quadruple mutations), and cultured in LB medium containing 100 mg/L ampicillin at 37 °C until optical density at 600 nm (OD_600_) reached 0.5–1.0. The expression of the GST-fused *Tc*PINK1^DDEE^ (GST-*Tc*PINK1^DDEE^) was induced by adding isopropyl-β-D-thiogalactopyranoside (IPTG) at the final concentration of 0.1 mM. The culture was further incubated for 18 h at 15 °C. The cells were collected by centrifugation and then disrupted by sonication in 50 mM Tris-HCl buffer (pH 7.2) containing 150 mM NaCl, 0.1% Triton X-100 and 1 mM DTT. GST-*Tc*PINK1^DDEE^ was purified by a Glutathione Sepharose FF (GE Healthcare) column. The GST tag of the eluted GST-*Tc*PINK1^DDEE^ was cleaved by HRV3C protease for 18 h at 4 °C. Lambda protein phosphatase was added to the HRV3C-treated sample (*i*.*e*., *Tc*PINK1^DDEE^) at the final concentration of 50 mg/L to dephosphorylate minor phosphorylated sites of *Tc*PINK1^DDEE^ and dialysed against 50 mM Tris-HCl buffer (pH 8.0) containing 1 mM MnCl_2_ for 18 h at 4 °C. The sample was further dialysed against 50 mM Tris-HCl (pH 8.0) buffer containing 1 mM DTT for at 3 h at 4 °C. The phosphatase-treated *Tc*PINK1^DDEE^ was purified by a ResourceQ anion exchange column (GE Healthcare) in 50 mM Tris-HCl buffer (pH 8.0) containing 1 mM DTT with a linear gradient of 0–300 mM NaCl. The purified *Tc*PINK1^DDEE^ was subjected to a HiLoad 16/60 Superdex200 prep grade (pg) size-exclusion column (GE Healthcare) in 10 mM Tris-HCl buffer (pH 7.2) containing 50 mM NaCl and 5 mM β-mercaptoethanol. The kinase activity of *Tc*PINK1^DDEE^ was tested by reacting 35 μM *Tc*PINK1^DDEE^ with 0.1 mM Ub in the kinase reaction buffer (50 mM Tris-HCl buffer (pH 7.2) containing 100 mM NaCl, 1 mM DTT, 10 mM ATP and 10 mM MgCl_2_) for 18 h at 32 °C. The fractions abundant in *Tc*PINK1^DDEE^ were collected and concentrated to approximately 4 g/L using Amicon Ultra-15 (Millipore). For the preparation of the selenomethionine (SeMet)-labeled sample, *E*. *coli* strain B834 (DE3) cells were transformed with the expression vector and cultured in minimal medium containing 10 g/L glucose, 100 mg/L ampicillin, 1 mM MgCl_2_, 1 mM MgSO_4_ and 50 g/L L-SeMet.

The His_6_-tagged lambda protein phosphatase was produced in *E*. *coli* strain Rosetta (DE3) cells (Invitrogen) and purified by a Ni-NTA Superflow column (Qiagen) in 50 mM Tris-HCl buffer (pH 8.0) containing 150 mM NaCl, 0.5% Triton X-100 and 20 mM (wash) or 200 mM (elution) imidazole. The purified phosphatase was concentrated to 10 g/L using Amicon Ultra-15 (Millipore), mixed with an equal volume of 90% glycerol, and stored at −30 °C until use.

### Crystallisation

*Tc*PINK1^DDEE^ was mixed with adenosine 5′-(β-γ-imido)triphosphate (AMP-PNP) (Sigma-Aldrich) and MgCl_2_ at the final concentrations of 1 mM. Initial crystallisation screening was performed using the sitting drop vapour diffusion method at 20 °C with a mosquito liquid-handling robot (TTP Lab Tech). More than 900 conditions of crystallisation reagent kits from Hampton Research, Qiagen and Molecular Dimensions were tested. The native or SeMet-labeled sample was mixed with an equal amount of the reservoir solution. The crystals of the native or SeMet-labeled *Tc*PINK1^DDEE^ were grown in 0.1 M HEPES-Na buffer (pH 7.0) containing 200 mM calcium acetate hydrate and 24% PEG400. For data collection, the crystals were soaked in the reservoir solution containing 15–30% glycerol and then flash frozen in liquid N_2_.

### Structure determination

Diffraction data sets of the native or SeMet-labeled *Tc*PINK1^DDEE^ were collected at beamline BL41XU in SPring-8 (Hyogo, Japan) and processed with HKL2000^42^ (HKL Research) and the CCP4 program suite^43^. The structure of the SeMet-labeled *Tc*PINK1^DDEE^ was solved by the single-wavelength anomalous dispersion method using the programs AutoSol and AutoBuild in the Phenix software suite^44^. The atomic model was corrected using the program Coot^45^ with careful inspection. Structure refinement was carried out using the program Phenix with iterative correction and refinement of the atomic model. The crystals of the SeMet-labeled *Tc*PINK1^DDEE^ diffracted better than those of the native *Tc*PINK1^DDEE^. We therefore used the data sets from the SeMet-labeled *Tc*PINK1^DDEE^ crystals for the structure refinement. Data collection and refinement statistics are shown in Supplementary Table 1. All molecular graphics were prepared with PyMOL (DeLano Scientific; http://www.pymol.org). The superposition of KMP onto the AMP-PNP-bound *Tc*PINK1 was computationally performed using PyMOL plugin. The coordinates of KMP for this superposition were derived from the crystal structure of the KMP-bound 2′-deoxynucleoside 5′-phosphate *N*-hydrolase (DNPH1; PDB 4KXN).

### *In vitro* kinase assay

*E*. *coli* strain pTf16/BL21 (TAKARA) cells were transformed with the expression vector for wild-type or mutant GST-*Tc*PINK1(128–570) and cultured in LB medium containing 100 mg/L ampicillin, 20 mg/L chloramphenicol and 0.5% L-arabinose at 37 °C until OD_660_ reached approximately 0.8. The expression was induced by IPTG at the final concentration of 0.1 mM for 18 h at 15 °C. The proteins were purified by a Glutathione Sepharose FF column and eluted with Tris-HCl buffer (pH 8.0) containing 150 mM NaCl, 1 mM DTT and 15 mM reduced glutathione. The concentration of the purified protein was calculated from the absorbance at 280 nm using Nanodrop 2000 (Thermo Fisher Scientific) and adjusted by dilution for each kinase assay. Wild-type or mutant *Tc*PINK1 (6.25 μM) was incubated with Ub (100 μM) in the kinase reaction buffer for 60 min at 32 °C. The term ‘1/10’ indicates that the amount of the enzyme is one-tenth of the control. The reaction was stopped by boiling for 5 min at 95 °C in 6× SDS sample buffer. The phosphorylated Ub was separated from the non-phosphorylated Ub by electrophoresis in 16 or 18% polyacrylamide gel containing 50 μM Phos-tag acrylamide (Wako chemicals) and 100 μM MnCl_2_ and stained with Coomassie brilliant blue (CBB).

### Site-specific photocrosslinking

For translating the amber codon in the target protein gene as *p*-benzoyl-phenylalanine (BPA), competent cells of *E*. *coli* BL21(DE3) and SoluBL21 (Genlantis) harbouring the pEVOL-pBpF vector were generated. The cells were transformed with the pColdI vector encoding His_6_-Ub with the amber mutation at a specific site and grown in LB medium containing 100 mg/L ampicillin and 20 mg/L chloramphenicol at 37 °C. When OD_660_ was reached approximately 0.8, IPTG, L-arabinose and BPA were added at the final concentrations of 0.1 mM, 1% and 1 mM, respectively, for 18 h at 15 °C. IPTG and L-arabinose induced the expressions of His_6_-Ub and the engineered aminocyl-tRNA synthetase–tRNA pair, respectively. The BPA-containing His_6_-Ub mutant (Ub-BPA) was purified by Ni-NTA Superflow (Qiagen) and Hiload 16/60 superdex75 pg columns (GE Healthcare). The incorporation sites of Ub-BPA were selected for photocrosslinking to *Tc*PINK1, following a previous photocrosslinking study on parkin^46^. GST-*Tc*PINK1 (10 μM) and Ub-BPA (30 μM) were mixed in the kinase reaction buffer and subject to UV irradiation at 365 nm using OmniCure S1500 (Lumen Dynamics) for 30 min on ice. The crosslinked products were separated by SDS-PAGE and detected by CBB.

### NMR analysis

For ^15^N labeling of Ub, 0.5 g/L ^15^NH_4_Cl was used as nitrogen source for M9 culture. Ub and *Tc*PINK1 for NMR analysis were purified in a manner similar to those for crystallisation. The final concentrations of Ub and *Tc*PINK1 were 50 μM and ~40 μM in a final volume of 250 μL of the NMR sample, respectively, which included 10 mM Tris-HCl (pH 7.5), 100 mM NaCl, 5 mM β-mercaptoethanol, 1 mM DTT, 5 mM AMP-PNP and 1 mM MgCl_2_ at 25 °C. The spectra were recorded at 600 MHz at ^1^H frequency. NMR experiments were performed at using a Bruker AVANCEIII 600 equipped with a TCI CRYOPROBE. All NMR spectra were processed using NMRPipe software^47^ and analysed using Sparky (Goddard, T. D. & Kneller, G. D.; https://www.cgl.ucsf.edu/home/sparky/).

### Mass spectrometry

The S205D S377D T386E triple mutant of His_6_-SUMO-*Tc*PINK1 was separated by Phos-tag SDS-PAGE and stained by CBB. The band of the phosphorylated His_6_-SUMO-*Tc*PINK1 triple mutant was cut and treated with 50% acetonitrile (ACN) in 100 mM ammonium bicarbonate (AMBC) twice for 30 min and 15 min and 100% ACN for 15 min. The dried gel pieces were incubated in 100 mM AMBC containing 10 mM DTT for 60 min at 56 °C. The reduced samples were incubated in 100 mM AMBC containing iodoacetamide for 45 min at room temperature. The alkylated samples were incubated in 100 mM AMBC for 10 min and treated with 100% ACN for 15 min twice. The dried gel pieces were incubated with 10 ng/μL Trypsin Gold (Promega) in 50 mM AMBC for 18 h at 37 °C. The peptides in the gel pieces were extracted using Ultrapure water (Wako Chemicals) for 20 min, 60% ACN and 0.1% TFA for 15 min, 80% ACN and 0.1% TFA for 15 min, 99.9% ACN and 0.1% TFA for 15 min. The solution containing the peptides were evaporated and applied to LC-MS/MS analysis system. NanoLC-MS/MS analysis was conducted by LTQ-Orbitrap Velos mass spectrometer (Thermo Fisher Scientific) equipped with a nanoLC interface (AMR), a nanoHPLC system (Michrom Paradigm MS2), and an HTC-PAL autosampler (CTC, Analytics). Mobile phases consisting of (A) 0.1% formic acid in Ultrapure water (Wako Chemicals) and (B) 100% acetonitrile. The in-gel digested samples were loaded onto a trap column (0.3 mm ID × 5 mm, 5 μm, L-column; CERI) and directly connected to a Zaplous α Pep-C18 packed column (3 μm, 0.1 × 150 mm) (AMR). The nanoLC gradient was delivered at 500 nL/min with a linear gradient of mobile phase B developed from 5 to 45% in 60 min. The mass spectrometer was operated in positive ionization mode and isolated charged ions were fragmented in the linear ion trap by collision-induced dissociation. The MS data were searched against the protein sequence database of *Tribolium castaneum* from Uniprot through the application of the search program Proteome Discoverer 1.4 (Thermo Fisher Scientific) for identification of the phosphorylated sites.

### *In cell* analysis

Wild-type and *PINK1*-knockout HeLa cells^48^ were cultured at 37 °C with 5% CO_2_ in Dulbecco’s modified Eagle medium (DMEM) containing 10% fetal bovine serum, penicillin-streptomycin, non-essential amino acids and sodium pyruvate (Life Technologies). The cells were transfected with Fugene6 (Promega) for 16–24 h. For immunoblotting analysis, the cells were lysed by TNE buffer (20 mM Tris-HCl buffer (pH 8.0) containing 150 mM NaCl, 1 mM EDTA-Na and 1% NP-40) supplemented with Protease Inhibitor Cocktail (Nacalai tesque). After centrifugation at 20,000 g for 10 min, the supernatant was subjected to immunoblotting. Anti-parkin antibody (Sigma-Aldrich, PRK8, 1:2,000) and anti-PINK1 antibody (Cell Signaling, D8G3, 1:1,000) were used as the primary antibodes. HRP-conjugated anti-mouse IgG antibody (MBL, product number 330, 1:5,000) and anti-rabbit IgG antibody (Santa Cruz Biotechnology, sc-2004, 1:5,000) were used as the secondary antibody.

### Data availability

The coordinates and structure factors of *Tc*PINK1^DDEE^ in complex with AMP-PNP have been deposited in the Protein Data Bank under the accession code 5YJ9. Other data are available from the corresponding author upon reasonable request.

## Electronic supplementary material

Supplementary Figures and Tables

Dataset 1
