# Supplementary material for: Structural insights into ubiquitin phosphorylation by PINK1
Source: Sci Rep. 2018 Jul 10;8:10382. doi: 10.1038/s41598-018-28656-8 (PMC6039469; doi:10.1038/s41598-018-28656-8)
Supplement: Supplementary file 1 — Supplementary Figures and Tables [file 41598_2018_28656_MOESM1_ESM.pdf]

Supplementary Information

Structural insights into ubiquitin phosphorylation by PINK1

Kei Okatsu[1,2], Yusuke Sato[1–3], Koji Yamano[4], Noriyuki Matsuda[4,5], Lumi Negishi[1], Akiko Takahashi[1], Atsushi Yamagata[1–3], Sakurako Goto-Ito[1,2], Masaki Mishima[6], Yutaka Ito[6], Toshihiko Oka[7], Keiji Tanaka[4] & Shuya Fukai[1–3,*]

[1]Institute for Quantitative Biosciences, The University of Tokyo, Tokyo 113-0032, Japan

[2]Synchrotron Radiation Research Organization, The University of Tokyo, Tokyo 113-0032, Japan

[3]Department of Computational Biology and Medical Sciences, Graduate School of Frontier Sciences, The University of Tokyo, Chiba 277-8561, Japan

[4]Tokyo Metropolitan Institute of Medical Science, Tokyo 156-8506, Japan

[5]PRESTO, Japan Science and Technology Agency, Saitama 332-0012, Japan

[6]Graduate School of Science & Engineering, Tokyo Metropolitan University, Tokyo 192-0397, Japan

[7]Department of Life Science, Rikkyo University, Tokyo 171-8501, Japan

[*]Correspondence should be addressed to S.F. (fukai@iam.u-tokyo.ac.jp)

Supplementary Table 1 Data collection and refinement statistics.

	*Tc*PINK1^{DDEE}–AMP-PNP
**Data collection**	
Space group	*C*2
Cell dimensions	
a, b, c (Å)	138.0, 59.8, 50.8
α, β, γ (°)	90.0, 101.8, 90.0
Resolution (Å)	50.0–2.53 (2.57–2.53)
R_{sym}	0.146 (0.545)
$I/\sigma(I)$	23.2 (1.95)
Completeness (%)	93.8 (94.0)
Redundancy	9.1 (5.6)
**Refinement**	
Resolution (Å)	50–2.5
No. reflections	12,624
R_{work} / R_{free}	0.229/0.247
No. atoms	
Protein	2,733
Ligand/ion	33
Water	3
Average B-factors (Å^2)	
Protein	72.4
Ligand/ion	74.0
Water	74.6
R.m.s. deviations	
Bond lengths (Å)	0.004
Bond angles (°)	1.04

Values in parentheses are for highest-resolution shell.

Supplementary Table 2 Pathogenic PINK1 mutations around αF.

*Hs*PINK1	Mutation	*Tc*PINK1	Position	Intramolecular interactions (*Tc*PINK1 residues)
Leu347	Pro	Leu322	αE	Leu462
Ile368	Asn	Leu343	β7 (C-spine)	Leu321, Ile417
Pro416	Arg	Pro391	APE motif	Trp412
Glu417	Gly	Glu392	APE motif	Arg470
Trp437	Arg	Trp412	αF	Leu388, Pro391, Phe428, Tyr439, Leu463
Gly440	Glu	Gly415	αF	Val459, Ile462, Ile463
Ile442	Thr	Ile417	αF (C-spine)	Leu321, Ile343
Leu489	Pro	Leu462	αH	Leu322

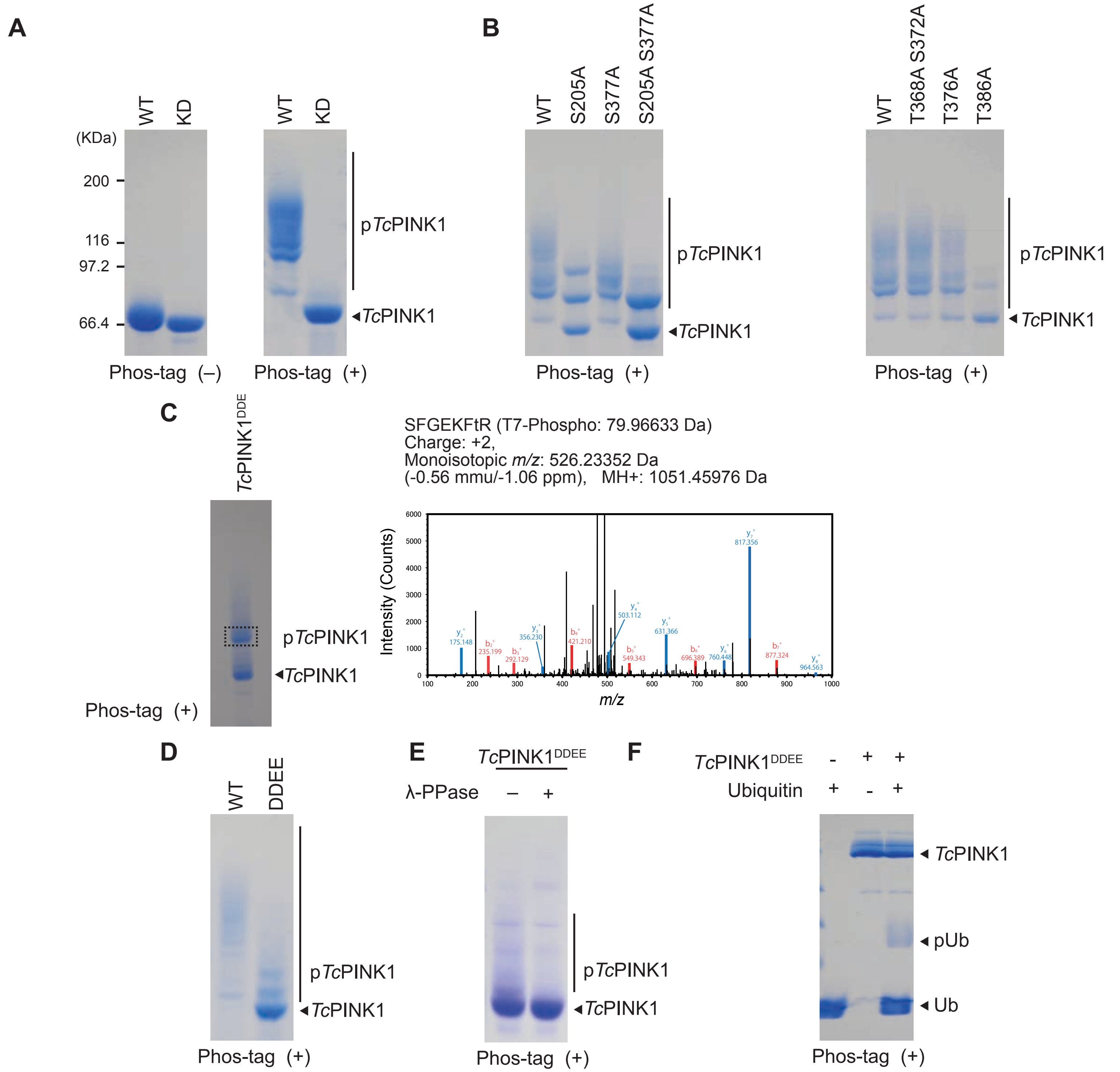

Supplementary Figure 1 Determination of the autophosphorylation sites in *Tc*PINK1.

Shown are the cropped images of gels stained with Coomassie brilliant blue. The contrast was adjusted for clarity. The uncropped images are shown in Supplementary Data 1.

(A) Autophosphorylation of wild-type (WT) and kinase-dead (KD) GST-*Tc*PINK1 proteins. The autophosphorylated products were analysed by SDS-PAGE with (+) or without (–) Phos-tag. Slowly migrated smear bands were detected only in Phos-tag gel, indicating the heterogeneous autophosphorylation of *Tc*PINK1 (p*Tc*PINK1).

(B) Autophosphorylation of GST-*Tc*PINK1 mutants at expected autophosphorylation sites. The autophosphorylated products were analysed by Phos-tag SDS-PAGE.

(C) Identification of major autophosphorylation sites of *Tc*PINK1. The phosphorylated S205D S307D T386E mutant of His_6-SUMO-*Tc*PINK1 (*Tc*PINK1^{DDE}) was separated by Phos-tag SDS-PAGE (left; enclosed by a dashed box) and subjected to LC-MS/MS analysis (right). The MS/MS data suggested that the autophosphorylation of *Tc*PINK1^{DDE} can occur at Thr530.

(D) Autophosphorylation of GST-*Tc*PINK1^{DDEE}. The autophosphorylated wild-type *Tc*PINK1 (WT) and *Tc*PINK1^{DDEE} (DDEE) were analysed by Phos-tag SDS-PAGE.

(E) Dephosphorylation of the autophosphorylated GST-*Tc*PINK1^{DDEE} by lambda protein phosphatase (λ-PPase). The intact and λ-PPase-treated samples were analysed by Phos-tag SDS-PAGE.

(F) Ub phosphorylation by *Tc*PINK1^{DDEE}. The phosphorylated products were analysed by Phos-tag SDS-PAGE.

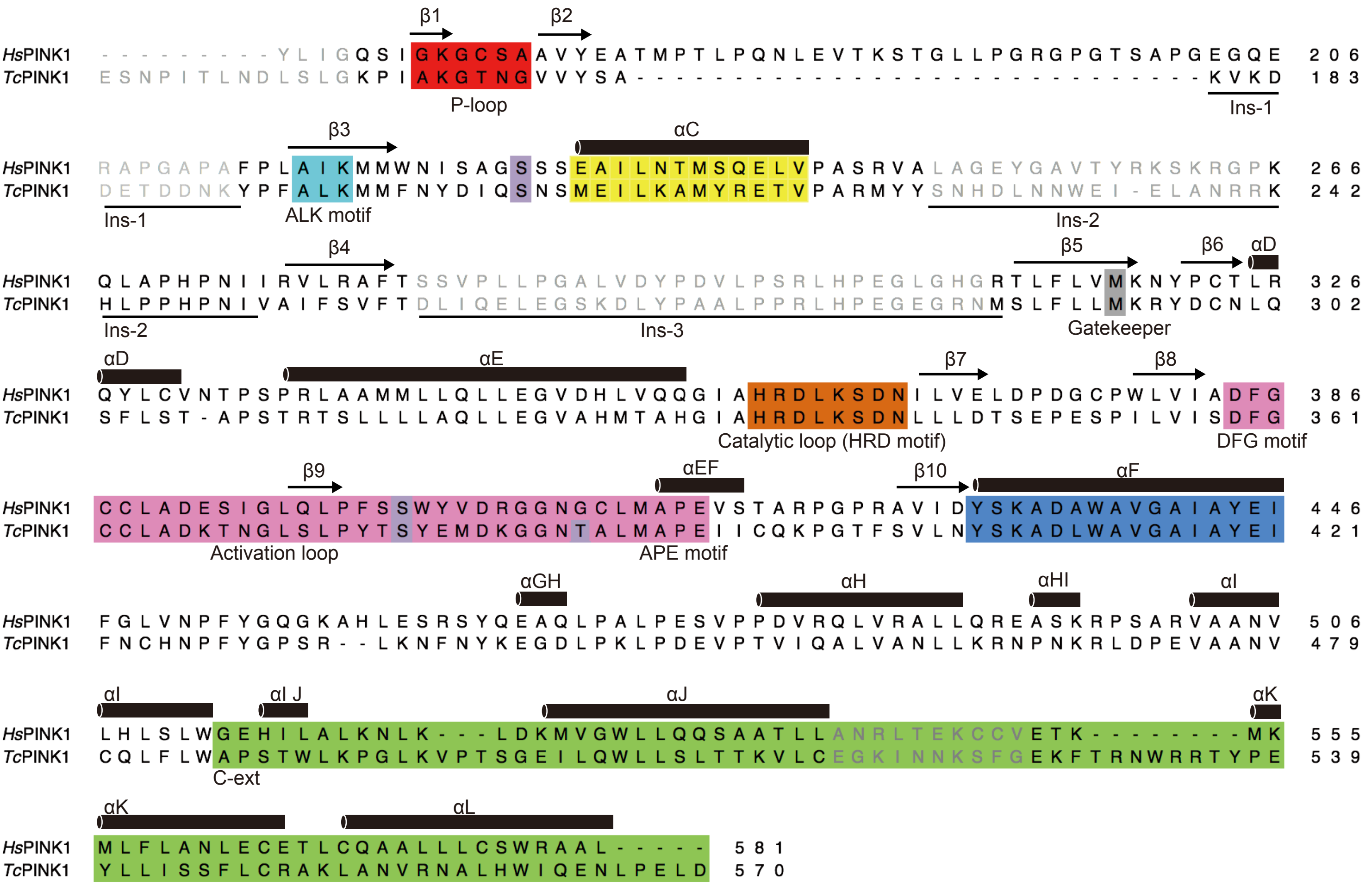

Supplementary Figure 2 Sequence alignment between *Hs*PINK1 and *Tc*PINK1.
The colouring scheme is as follows: P-loop, red; ALK motif, cyan; αC, yellow; gatekeeper, grey; catalytic loop, orange; activation loop, pink; αF, blue; C-ext, green; autophosphorylation sites, purple. The secondary structure of *Tc*PINK1^{DDEE} is shown above the alignment. The arrows and cylinders correspond to β-strands and α-helices, respectively. The black lines below the alignment indicate the PINK1-specific insertions. The disordered regions in the present *Tc*PINK1^{DDEE} structure are indicated by grey characters.

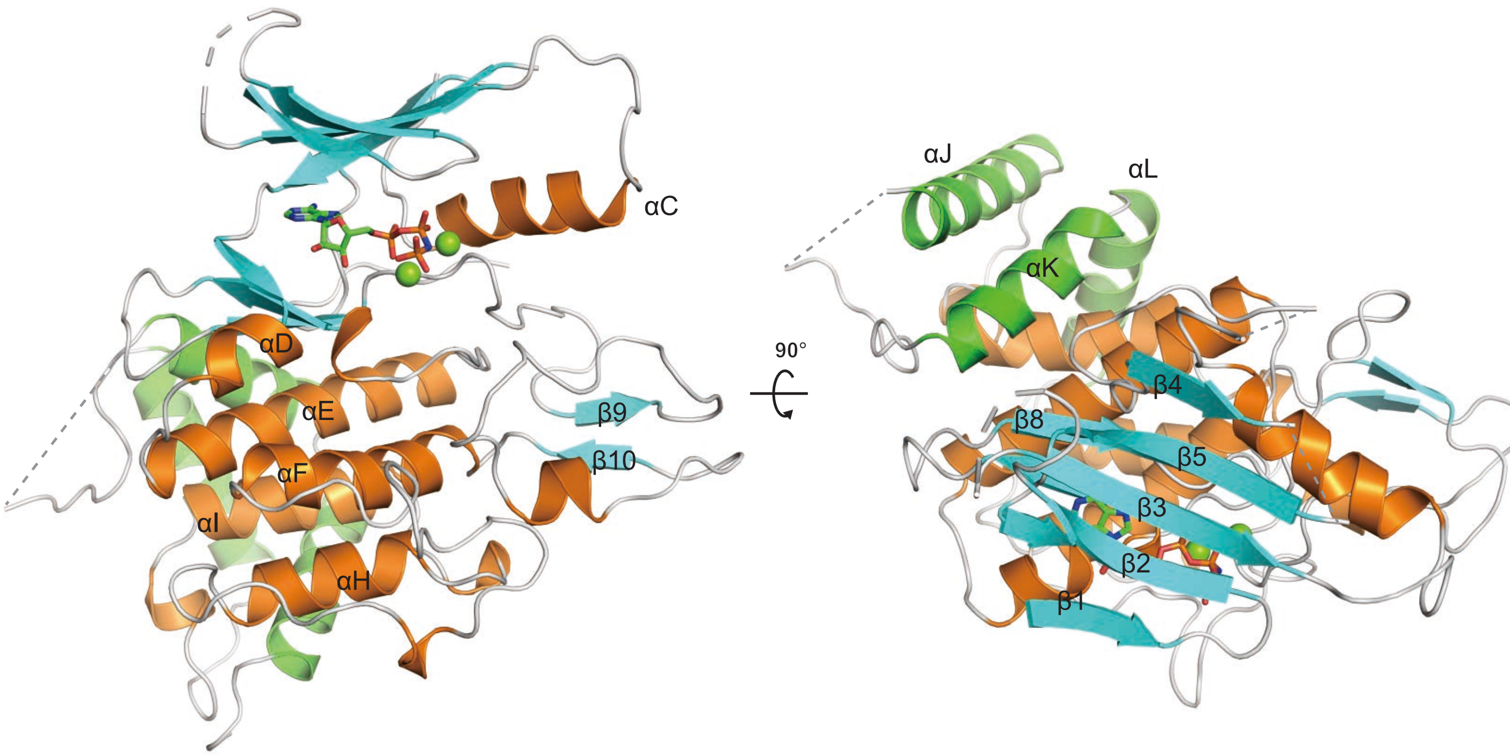

Supplementary Figure 3 Annotation of the secondary structure of *Tc*PINK1^{DDEE}.
β-strands and α-helices are coloured in cyan and orange, respectively, except that the α-helices in the C-ext was coloured in green. The bound ATP analogue is shown as sticks. The two coordinated Mg ions are shown as green spheres.

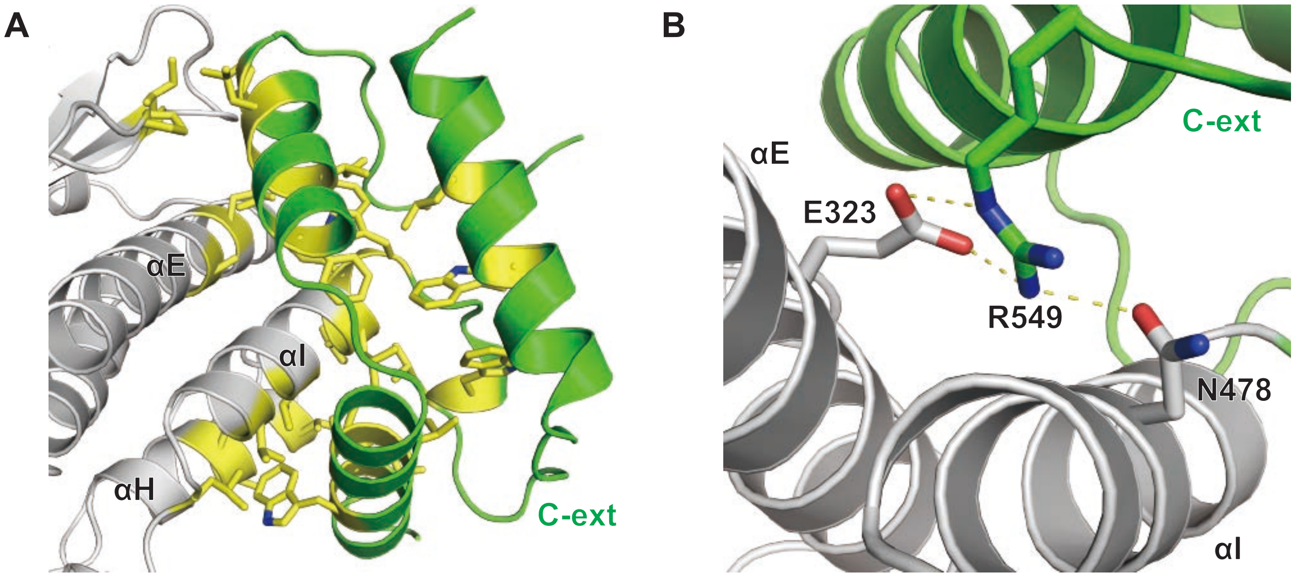

Supplementary Figure 4 Interactions between the C-lobe and C-ext in *Tc*PINK1^{DDEE}.
The colouring scheme is the same as that in Fig. 1A.
(A) Hydrophobic interactions between the C-lobe and C-ext. The residues involved in these interactions are shown as yellow sticks.
(B) Hydrophilic interactions between the C-lobe and C-ext. The residues involved in these interactions are shown as sticks.

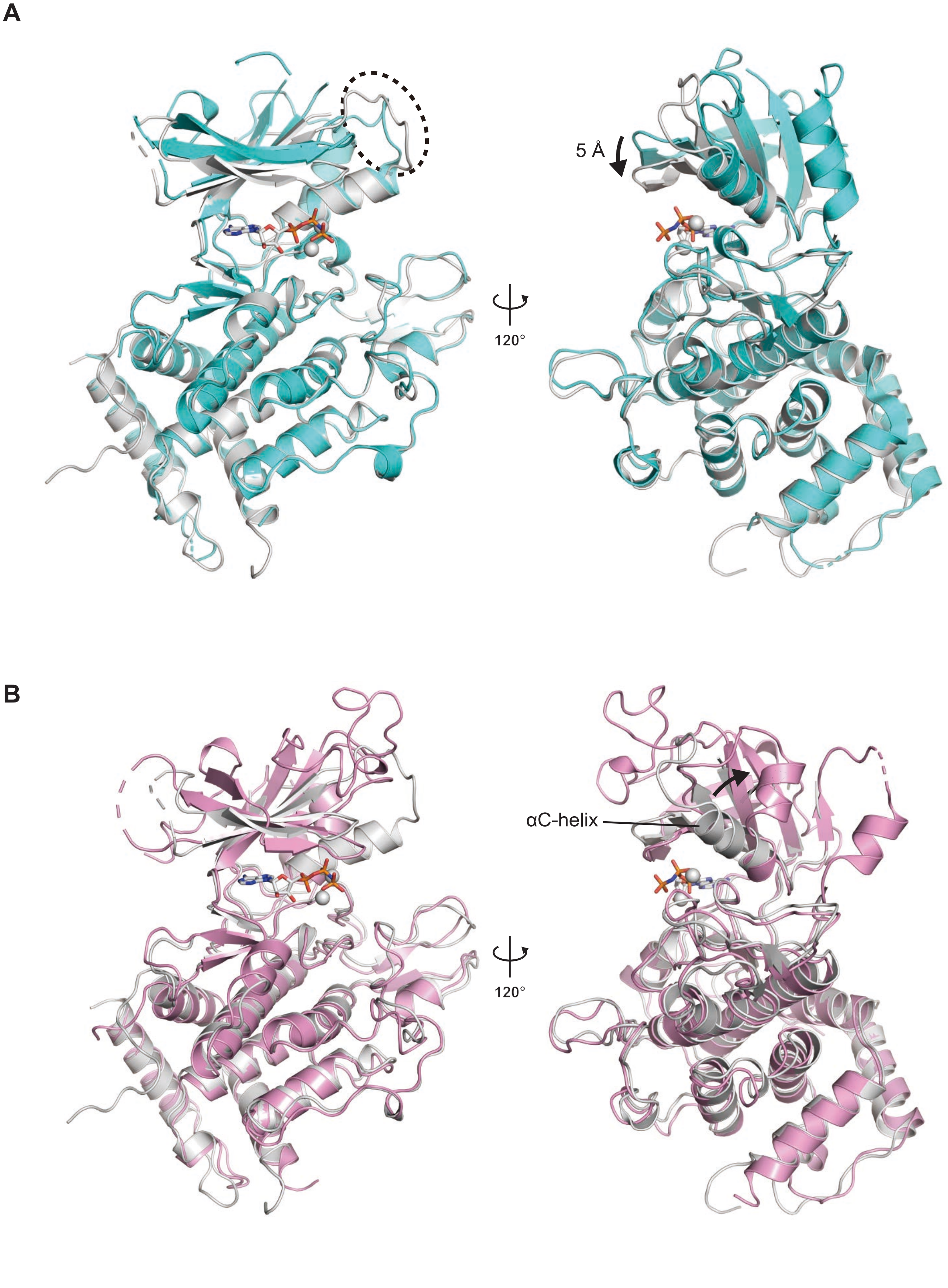

Supplementary Figure 5 Structural comparison of the AMP-PNP-bound *Tc*PINK1^{DDEE} with apo-*Tc*PINK1 or the Ub^{TVNL}-bound *Ph*PINK1.

The bound AMP-PNP and Mg ions are shown as sticks and spheres, respectively.

(A) Superposition of the AMP-PNP-bound *Tc*PINK1^{DDEE} (this study; white) and apo-*Tc*PINK1 (PDB 5OAT; cyan). The arrow in the right panel indicates that the N-lobe is shifted by approximately 5 Å (estimated from the Cα–Cα distance between Thr172 of the AMP-PNP-bound *Tc*PINK1^{DDEE} and Thr172 of apo-*Tc*PINK1 in the superposition). The flexible loop between β3 and αC is encircled by a dashed line.

(B) Superposition of the AMP-PNP-bound *Tc*PINK1^{DDEE} (this study; white) and Ub^{TVNL}-bound *Ph*PINK1 (PDB 6EQI; pink). The arrow in the right panel indicates that the flexible loop and αC are rearranged.

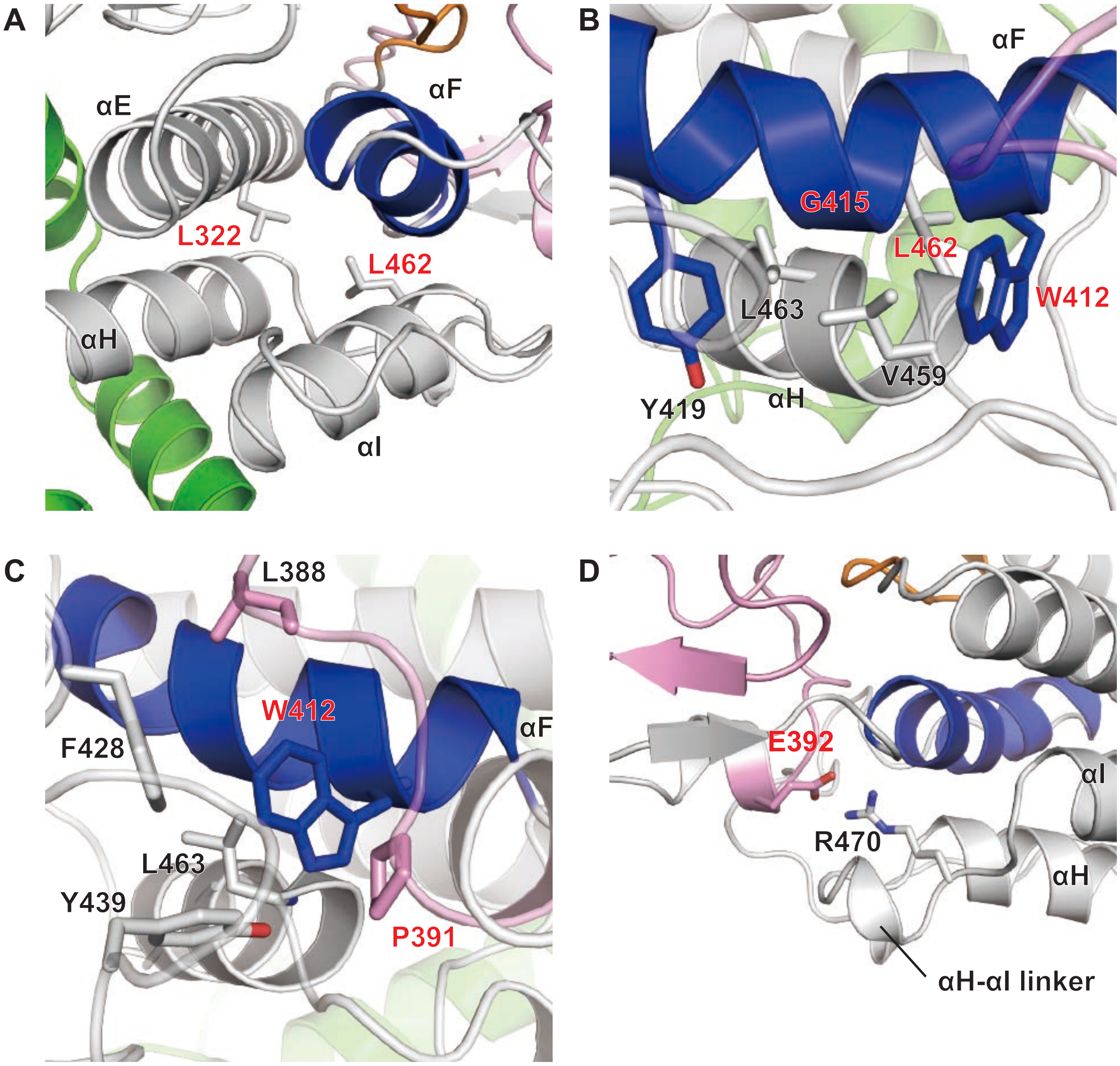

Supplementary Figure 6 Pathogenic mutation sites of PINK1.
*Tc*PINK1 residues corresponding to the reported pathogenic mutation sites of *Hs*PINK1 (highlighted by red characters) are shown as sticks. Neighbouring residues for the intramolecular interactions are also shown as sticks. The colouring scheme is the same as that in Fig. 1A.
(A) Close-up view of the area around Leu322 and Leu462.
(B) Close-up view of the area around Trp412, Gly415 and Leu462
(C) Close-up view of the area around Pro391 and Trp412
(D) Close-up view of the area around Glu392.

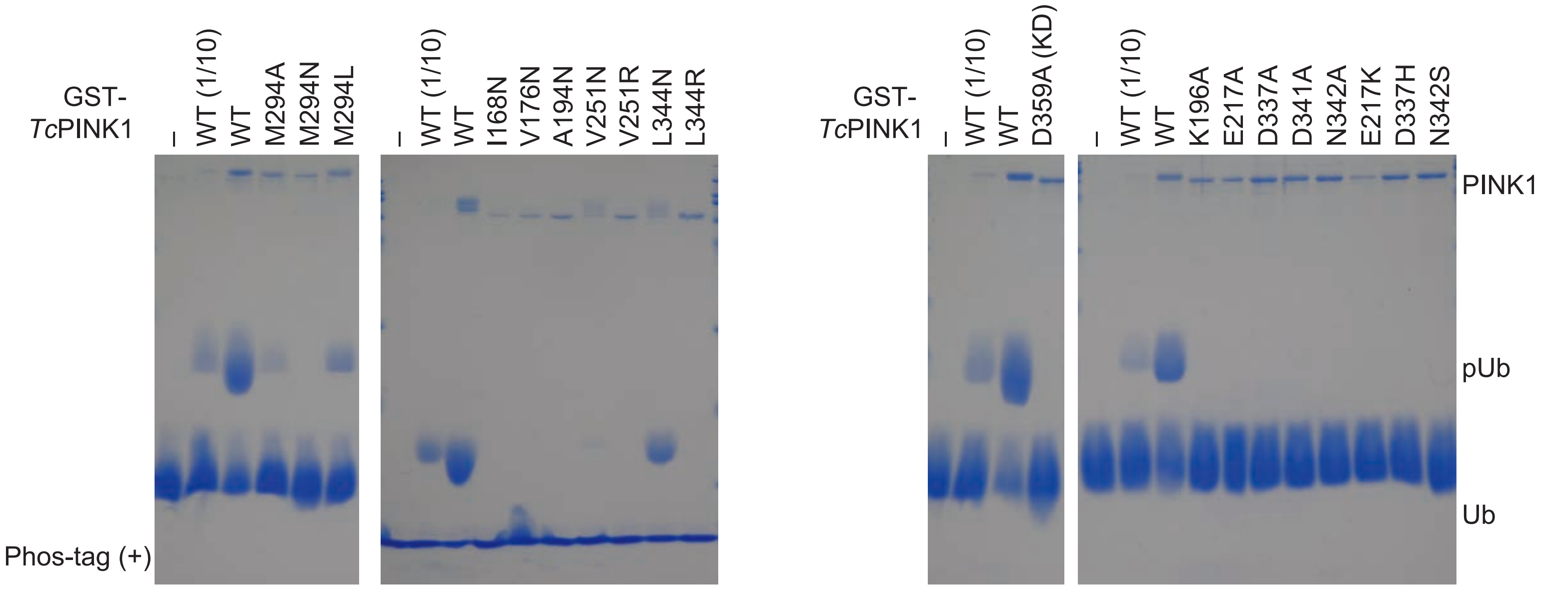

Supplementary Figure 7 Additional data of the experiments for Figures 2B and D
In vitro kinase assay of *Tc*PINK1 mutants that were designed to compromise the interaction with the adenine ring of ATP or coordination of the catalytic Mg ions was repeated. Ub phosphorylation (pUb) by GST-*Tc*PINK1 was analysed by Phos-tag SDS-PAGE. Shown are the cropped images of gels stained with Coomassie brilliant blue. The contrast was adjusted for clarity. The uncropped images are shown in Supplementary Data 1.

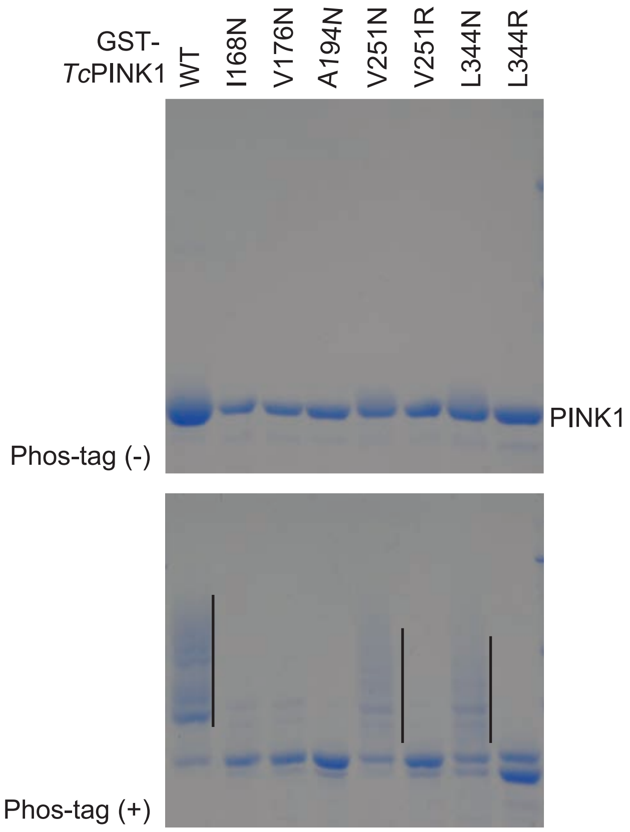

Supplementary Figure 8 Autophosphorylation of *Tc*PINK1 mutants in the hydrophobic pocket for recognition of the adenine ring. Autophosphorylation of wild-type (WT) and mutant GST-*Tc*PINK1 proteins. The autophosphorylated products were analysed by SDS-PAGE with (+) or without (–) Phos-tag. Slowly migrated smear bands were detected only in Phos-tag (+), indicating that *Tc*PINK1 was autophosphorylated heterogeneously. The lines indicate multiple phosphorylated species. Shown are the cropped images of gels stained with Coomassie brilliant blue. The contrast was adjusted for clarity. The uncropped images are shown in Supplementary Data 1.

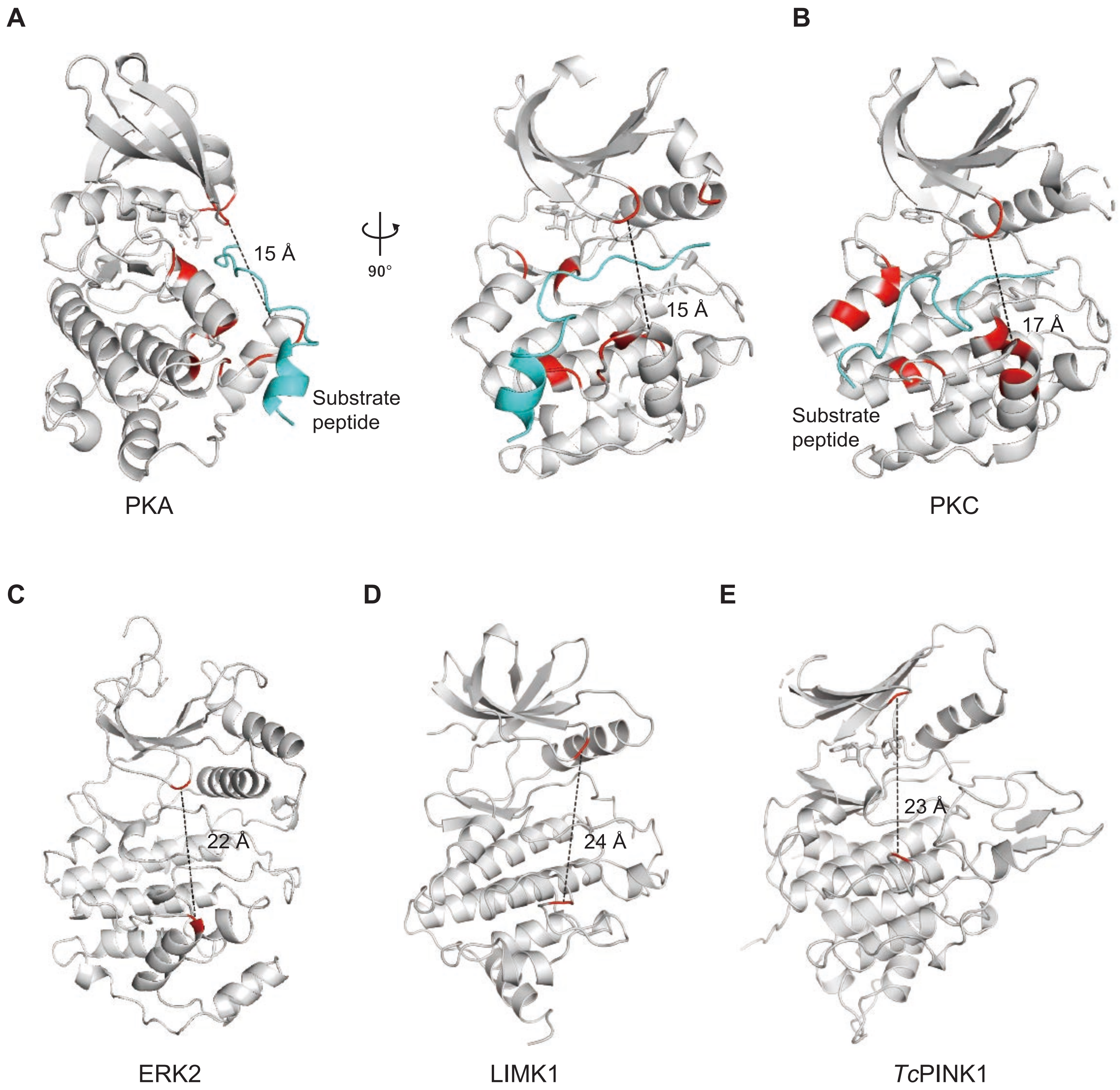

Supplementary Figure 9 Substrate-binding grooves of protein kinases

(A) Structure of the kinase domain of PKA (PDB 1ATP; residues 40–299) in complex with the substrate peptide. The substrate peptide is coloured in cyan, whereas the substrate peptide-interacting residues are coloured in red. The width of the substrate peptide-binding groove is estimated from the Cα–Cα distance between Ser53 and Pro243 (indicated by a dashed line).

(B) Structure of the kinase domain of PKC (PDB 4DC2; residues 239–511) in complex with the substrate peptide. The width of the substrate peptide-binding groove is estimated from the Cα–Cα distance between Ser254 and Glu457 (indicated by a dashed line). The colouring scheme is the same as that in (A).

(C) Structure of the kinase domain of ERK2 (PDB 2ERK; residues 6–358). The width of the substrate-binding groove is estimated from the Cα–Cα distance between Ala33 and Tyr231 (indicated by a dashed line). Ala33 and Tyr231 are coloured in red.

(D) Structure of the kinase domain of LIM (PDB 5HVK; residues 324–634). The width of the substrate-binding groove is estimated from the Cα–Cα distance between Cys349 and Asp551 (indicated by a dashed line). Cys349 and Asp551 are coloured in red.

(E) Structure of *Tc*PINK1^{DDEE}. The width of the putative Ub-binding groove is estimated from the Cα–Cα distance between Thr172 and Tyr429 (indicated by a dashed line). Thr172 and Tyr429 are coloured in red.

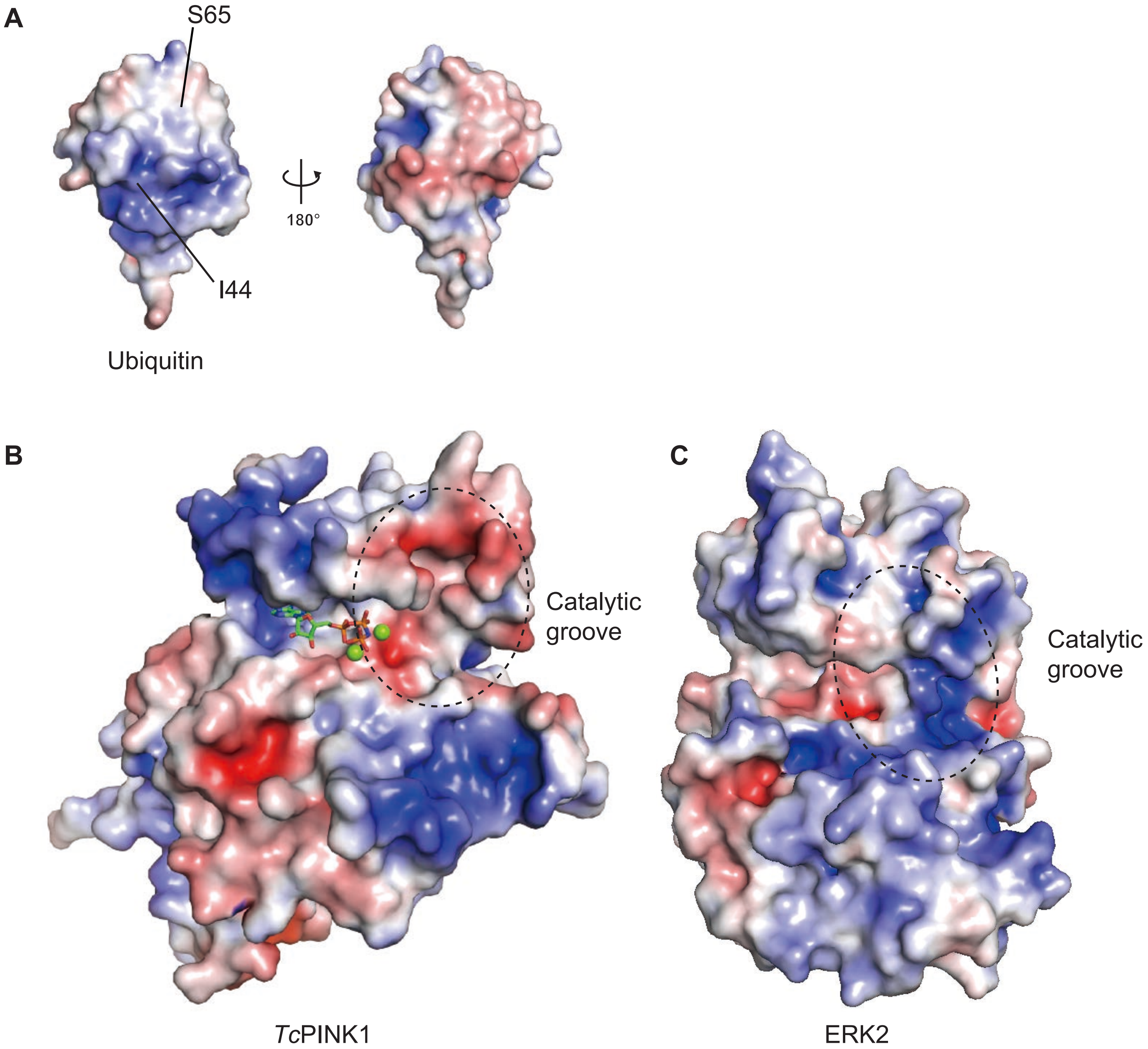

Supplementary Figure 10 Electrostatic surface potentials.
Electrostatic surface potentials were calculated at pH 7.5 by the programs APBS. The positive and negative charges are shown in blue and red, respectively, contoured from +5 to −5 $k_B T/e$.
(A) Electrostatic surface potential of Ub. The β-sheet side centered Ile44 (left) is more positively charged than the α-helix side (right).
(B) Electrostatics surface potential of *Tc*PINK1^{DDEE}. The putative Ub-binding groove (encircled by a dotted line) is negatively charged.
(C) Electrostatics surface potential of the kinase domain of ERK2. The substrate-binding groove (encircled by a dotted line) is positively charged.

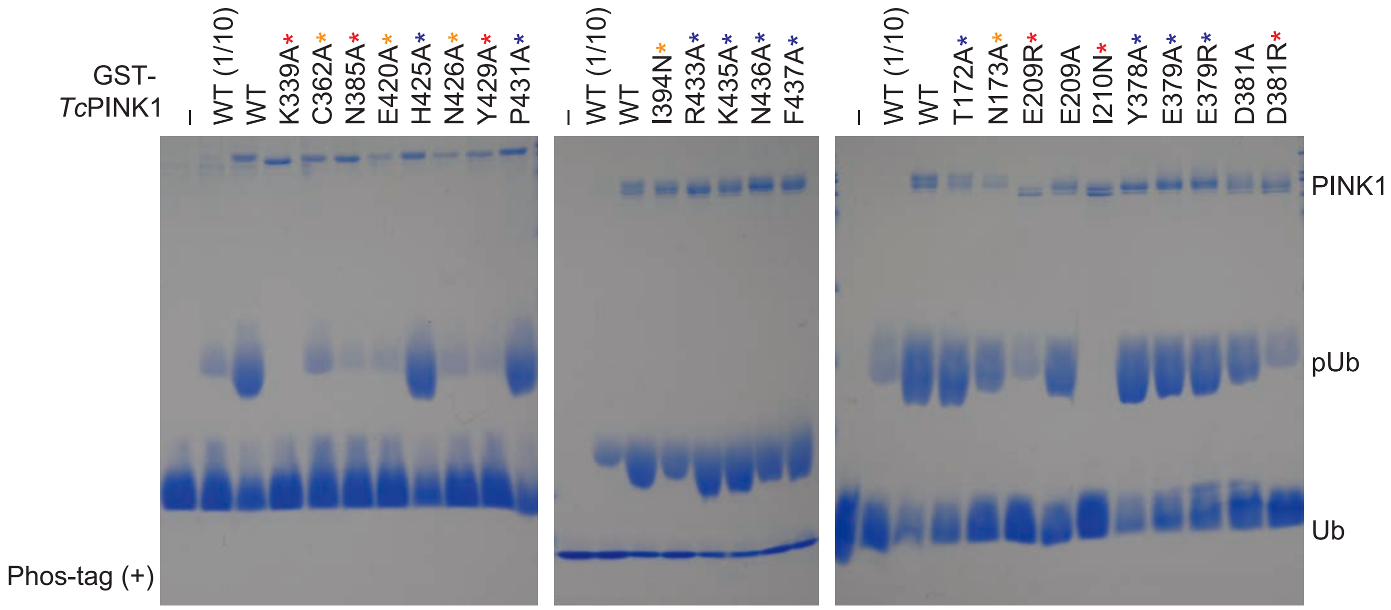

Supplementary Figure 11 Additional data of the experiment for Figure 3
In vitro kinase assay of *Tc*PINK1 mutants that were expected to compromise the interaction with Ub was repeated. Ub phosphorylation (pUb) by GST-*Tc*PINK1 was analysed by Phos-tag SDS-PAGE. Shown are the cropped images of gels stained with Coomassie brilliant blue. The contrast was adjusted for clarity. The uncropped images are shown in Supplementary Data 1.

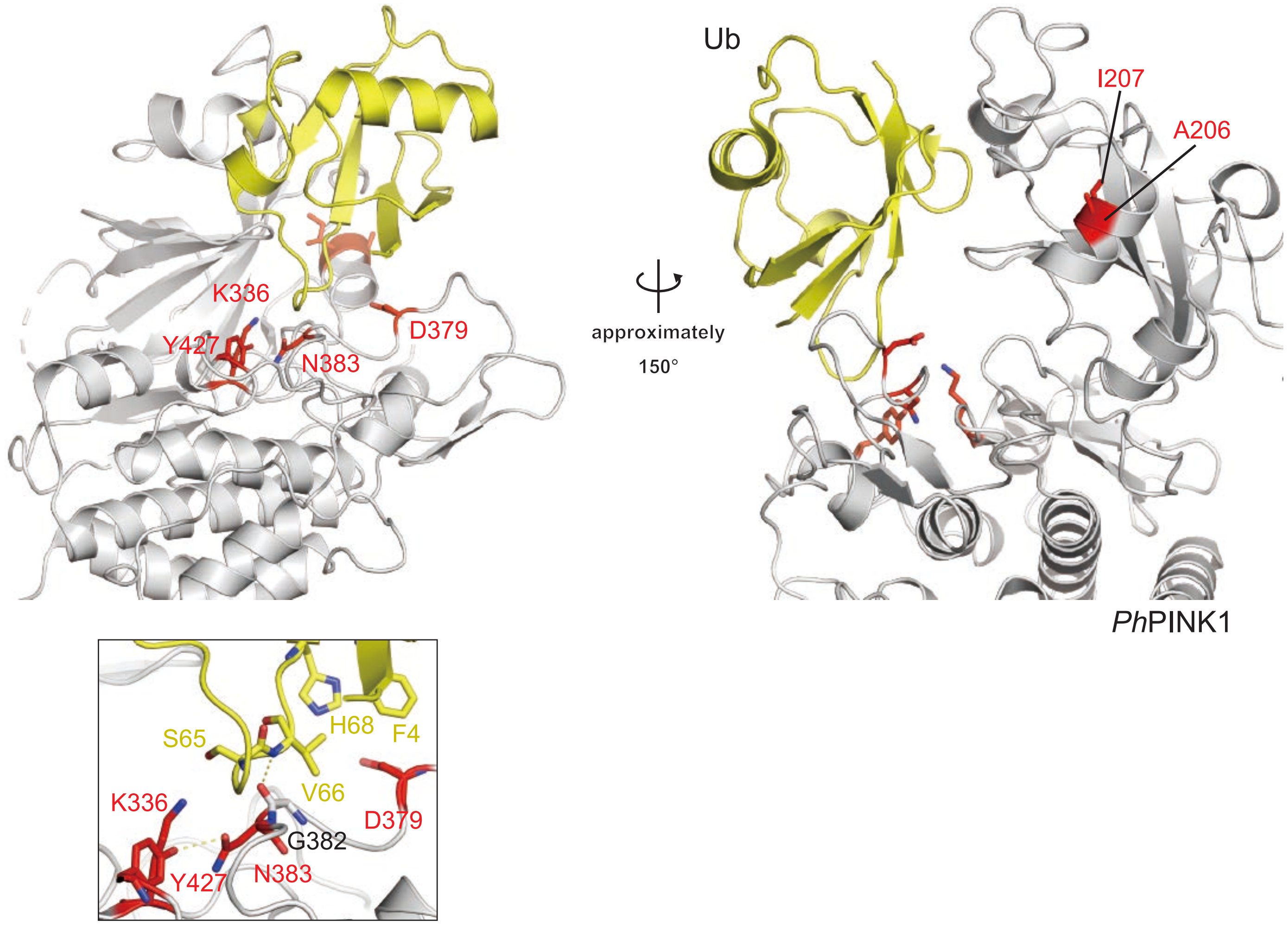

Supplementary Figure 12 Assessment of the potential Ub-interacting residues suggested by *in vitro* kinase assay of *Tc*PINK1 mutants.

Ala206, Ile207, Lys336, Asp379, Asn383 and Tyr427 of *Ph*PINK1, which correspond to Glu209, Ile210, Lys339, Asp381, Asn385 and Tyr429 of *Tc*PINK1, respectively, are shown as red sticks in the $\mathrm{Ub}^{\mathrm{TVNL}}$-bound *Ph*PINK1 structure (PDB 6EQI). $\mathrm{Ub}^{\mathrm{TVNL}}$ is coloured in yellow. A close-up view of the area around Lys336, Asp379, Asn383 and Tyr427 is also shown in the box.

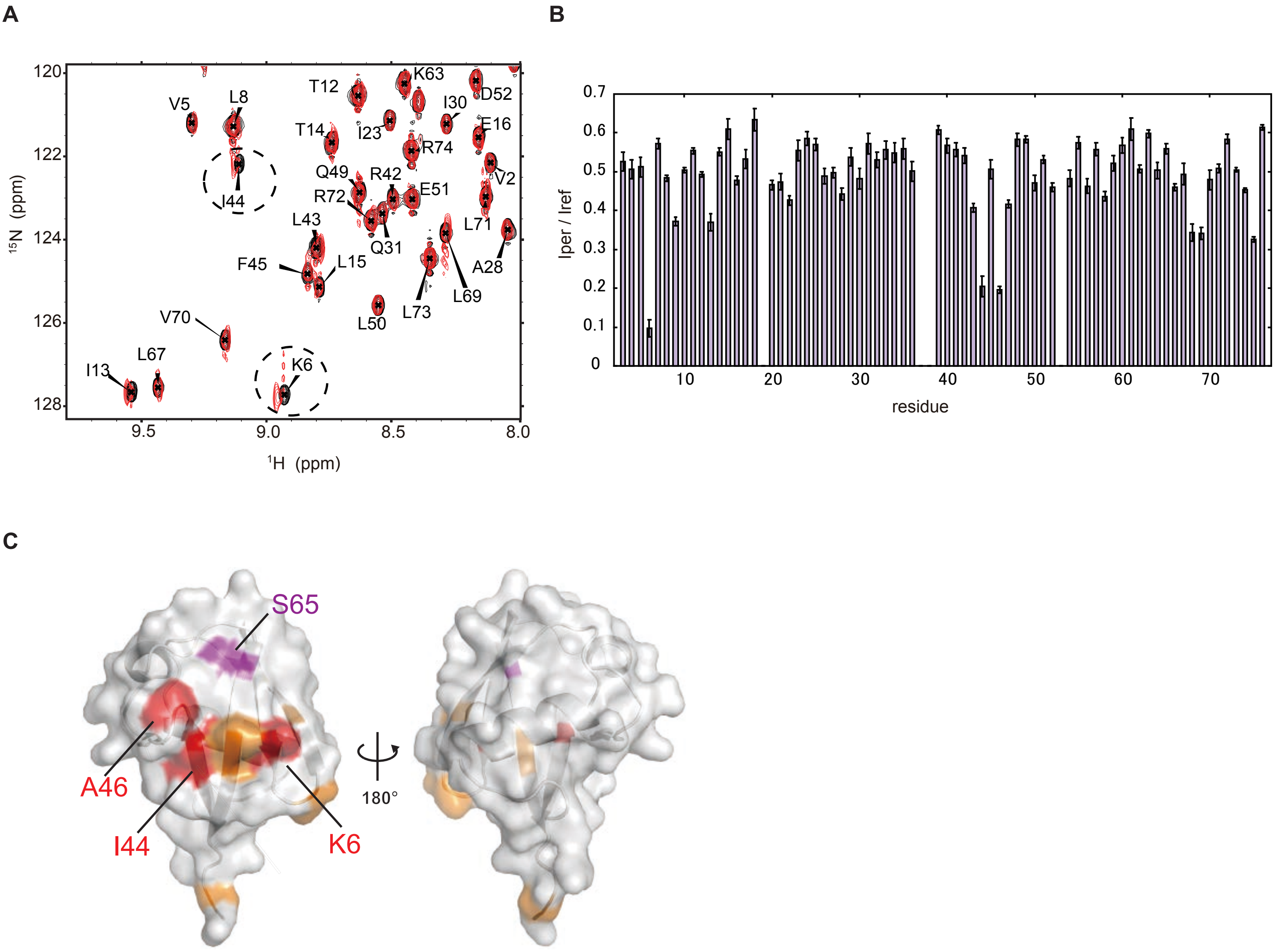

Supplementary Figure 13 NMR analysis.

(A) A selected region of the overlay of 2D ^{1}H–^{15}N HSQC spectra of the ^{2}H,^{15}N-labeled Ub in the presence (red) and in the absence (black) of *Tc*PINK1. The representative signals drastically perturbed are marked by dotted circle. Both samples included 1 mM Mg^{2+} and 5 mM AMP-PNP.

(B) Signal reduction versus amino acid residue for Ub. The data are represented by the intensity ratio I_{per}/I_{ref}, where I_{per} and I_{ref} were measured in the presence and in the absence of *Tc*PINK1, respectively. The error bars were calculated based on the signal-to-noise ratios.

(C) Potential *Tc*PINK1-interacting residues suggested by the NMR experiments. The potential *Tc*PINK1-interacting residues are mapped on the surface of Ub. The colour-coding is based on the intensity ratio I_{per}/I_{ref} for each residue in (B): red, drastic (less than or equal to 0.20); orange, mild (less than or equal to 0.40). Ser65 (purple) is also mapped.

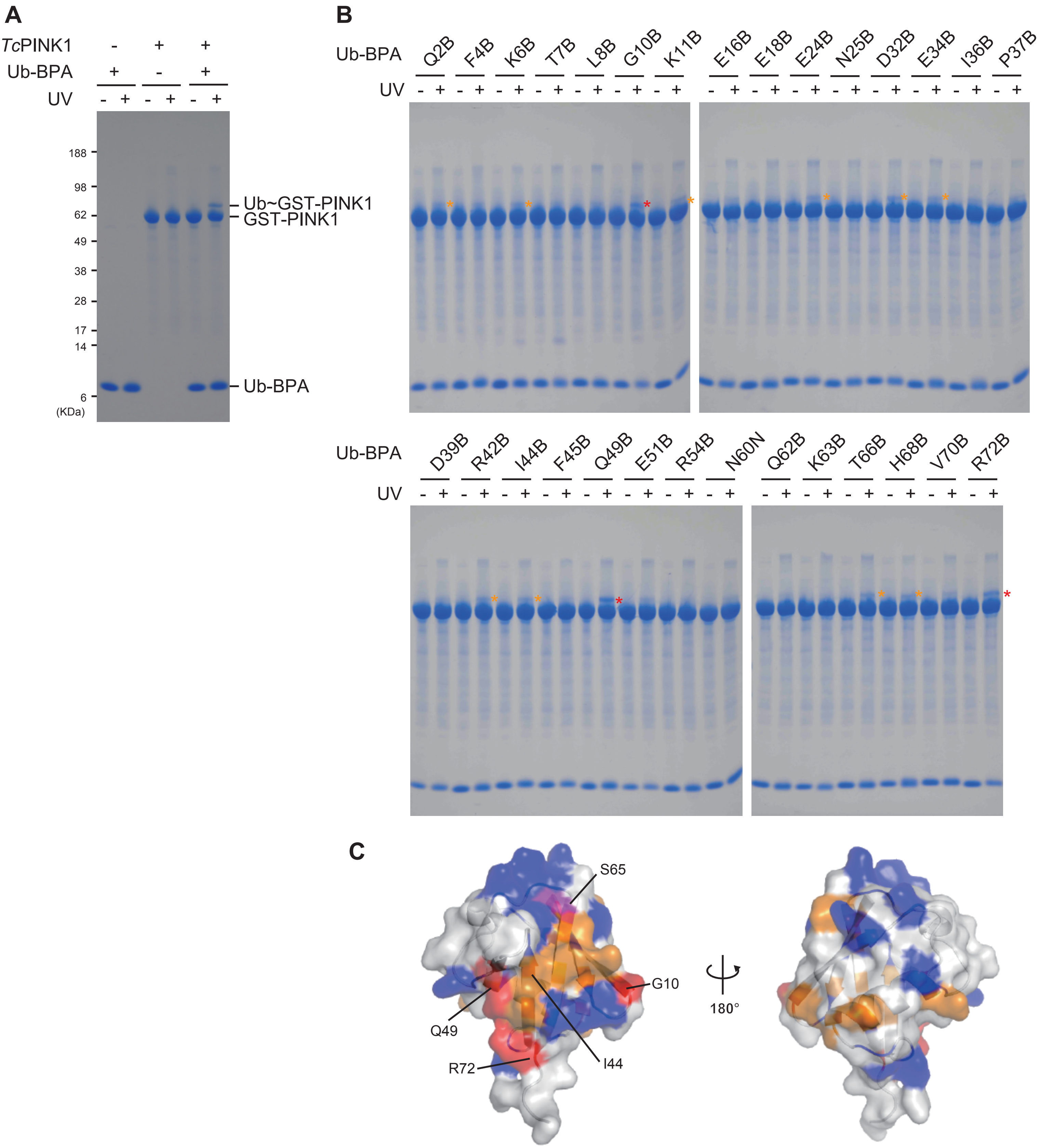

Supplementary Figure 14 Photocrosslinking experiments between Ub and *Tc*PINK1.

Shown are the cropped gel images of gels stained with Coomassie brilliant blue. The contrast was adjusted for clarity. The uncropped images are shown in Supplementary Data 1.

(A) Photocrosslinking of His$_6$-Ub (Gln49BPA; Q49B) to GST-*Tc*PINK1. GST-*Tc*PINK1, His$_6$-Ub (Q49B) and their mixture were incubated in the kinase reaction buffer (see Materials and methods) with or without UV irradiation. The reaction mixtures were analysed by SDS-PAGE with Coomassie brilliant blue staining. The photocrosslinked product (Ub~GST-*Tc*PINK1) was detected only when the mixture was irradiated by UV.

(B) Photocrosslinking analysis of BPA-containing His$_6$-Ub mutants (Ub-BPA) with GST-*Tc*PINK1. BPA was comprehensively introduced to His$_6$-Ub in a site-specific manner and reacted to GST-*Tc*PINK1 with or without UV irradiation. The reaction mixtures were analysed by SDS-PAGE. The substitution of BPA for Gly10, Gln49 or Arg72 generated the clear band of the crosslinked product (red asterisks), whereas that for Gln2, Lys6, Lys11, Glu24, Asp32, Glu34, Arg42, Ile44, Thr66, or His68 generated the weak band (orange asterisks).

(C) Crosslinked residues mapped on the surface of Ub. Gly10, Gln49 and Arg72 are coloured in red, whereas Gln2, Lys6, Lys11, Glu24, Asp32, Glu34, Arg42, Ile44, Thr66, and His68 are coloured in orange. The other non-crosslinked residues are coloured in blue. Ser65 (the phosphorylation site) is coloured in purple.

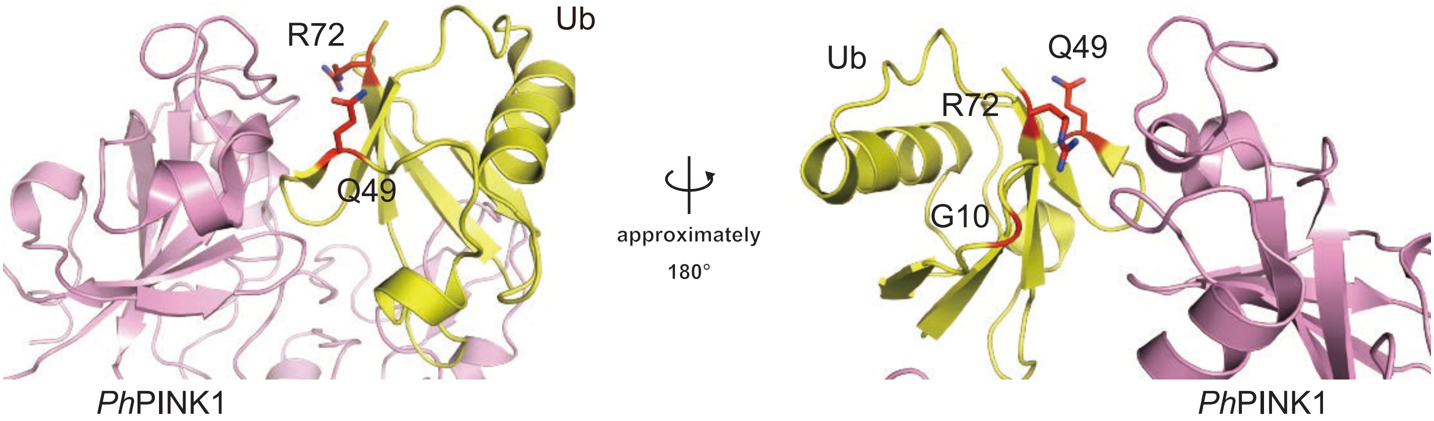

Supplementary Figure 15 Crosslinked sites mapped on the complex between *Ph*PINK1 and Ub^{TVNL}.
Ub^{TVNL} and *Ph*PINK1 are coloured in yellow and pink, respectively. Gly10, Gln49 and Arg72 (the major crosslinked sites shown in Supplementary Fig. 14) are shown as red sticks.

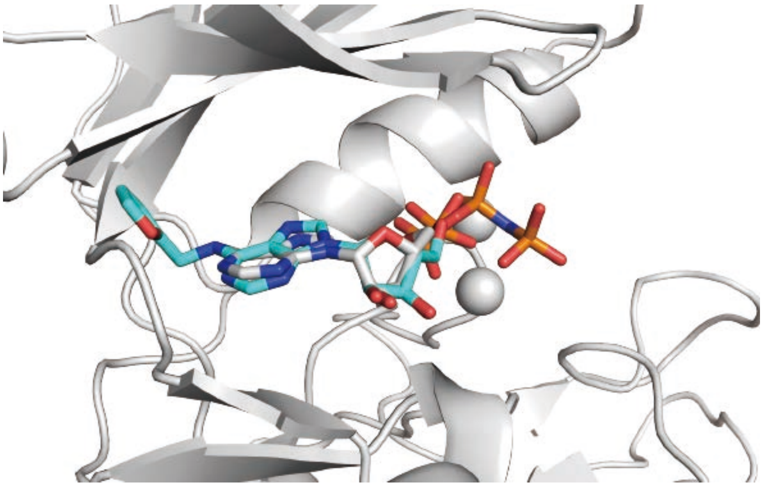

Supplementary Figure 16 Superposition of KMP onto the AMP-PNP-bound *Tc*PINK1^{DDEE}.
The AMP-PNP and KMP molecules are shown as sticks. The carbon atoms of AMP-PNP and KMP are coloured in white and cyan, respectively. The coordinated Mg ions are shown as white spheres. *Tc*PINK1^{DDEE} is coloured in white.
