# Supplementary material for: Structural insights into ubiquitin phosphorylation by PINK1
Source: Sci Rep. 2018 Jul 10;8:10382. doi: 10.1038/s41598-018-28656-8 (PMC6039469; doi:10.1038/s41598-018-28656-8)

**Supplementary Data**

**Structural insights into ubiquitin phosphorylation by PINK1**

Kei Okatsu^1,2^, Yusuke Sato^1–3^, Koji Yamano^4^, Noriyuki Matsuda^4,5^, Lumi Negishi^1^, Akiko Takahashi^1^, Atsushi Yamagata^1–3^, Sakurako Goto-Ito^1,2^, Masaki Mishima^6^, Yutaka Ito^6^, Toshihiko Oka^7^, Keiji Tanaka^4^ & Shuya Fukai^1–3,*^

^1^Institute for Quantitative Biosciences, The University of Tokyo, Tokyo 113-0032, Japan

^2^Synchrotron Radiation Research Organization, The University of Tokyo, Tokyo 113-0032, Japan

^3^Department of Computational Biology and Medical Sciences, Graduate School of Frontier Sciences, The University of Tokyo, Chiba 277-8561, Japan

^4^Tokyo Metropolitan Institute of Medical Science, Tokyo 156-8506, Japan

^5^PRESTO, Japan Science and Technology Agency, Saitama 332-0012, Japan

^6^Graduate School of Science & Engineering, Tokyo Metropolitan University, Tokyo 192-0397, Japan

^7^Department of Life Science, Rikkyo University, Tokyo 171-8501, Japan

^*^Correspondence should be addressed to S.F. (fukai@iam.u-tokyo.ac.jp)


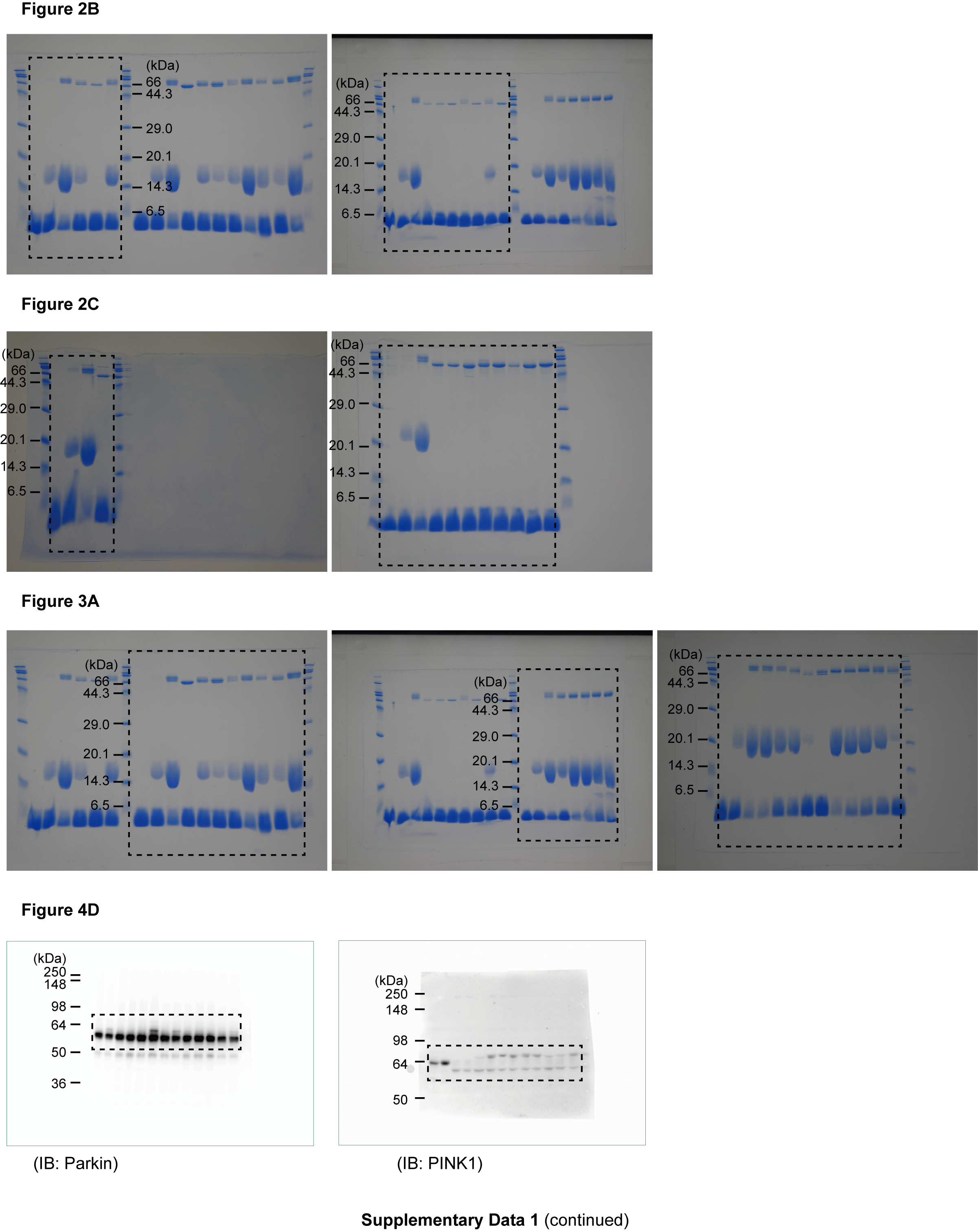


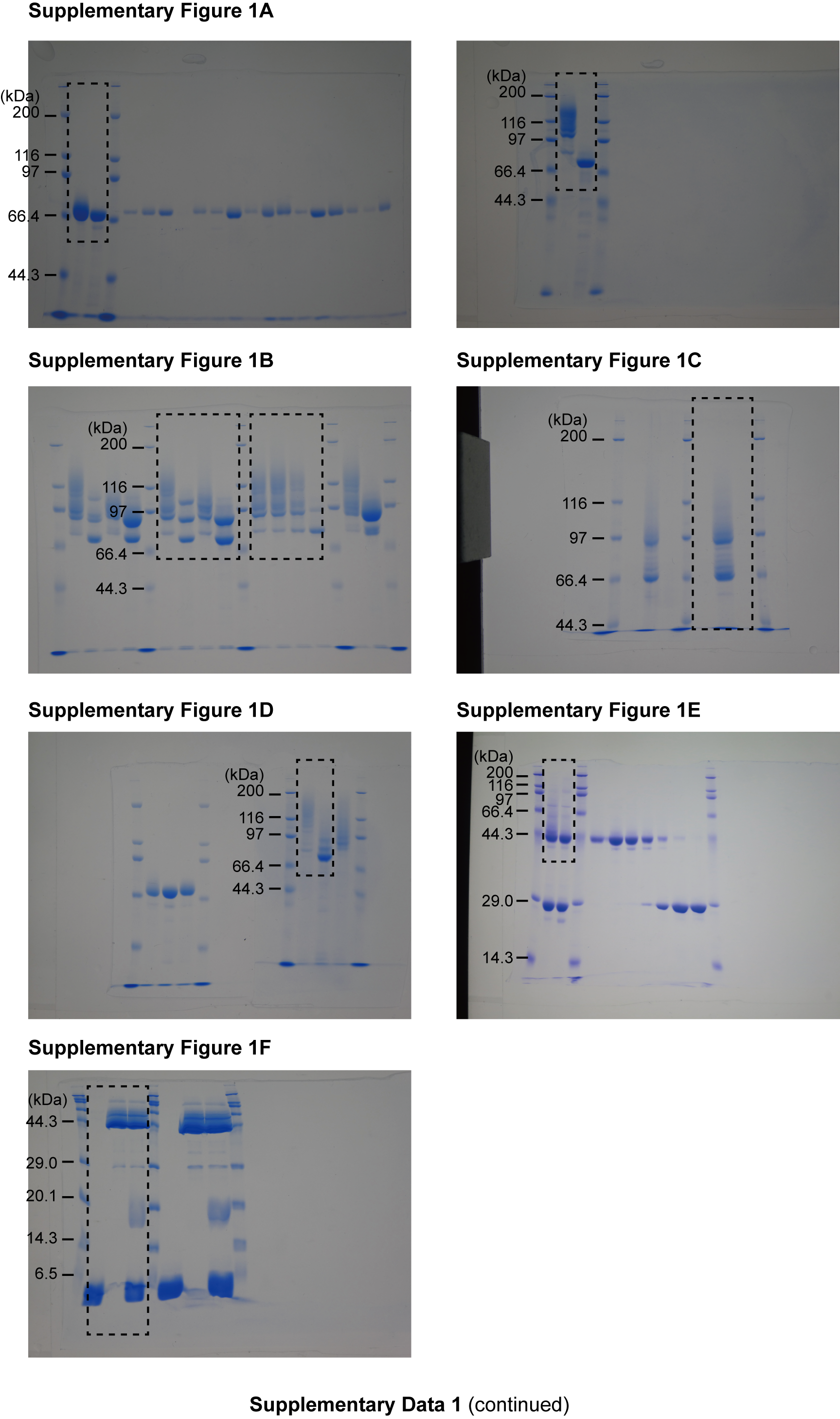


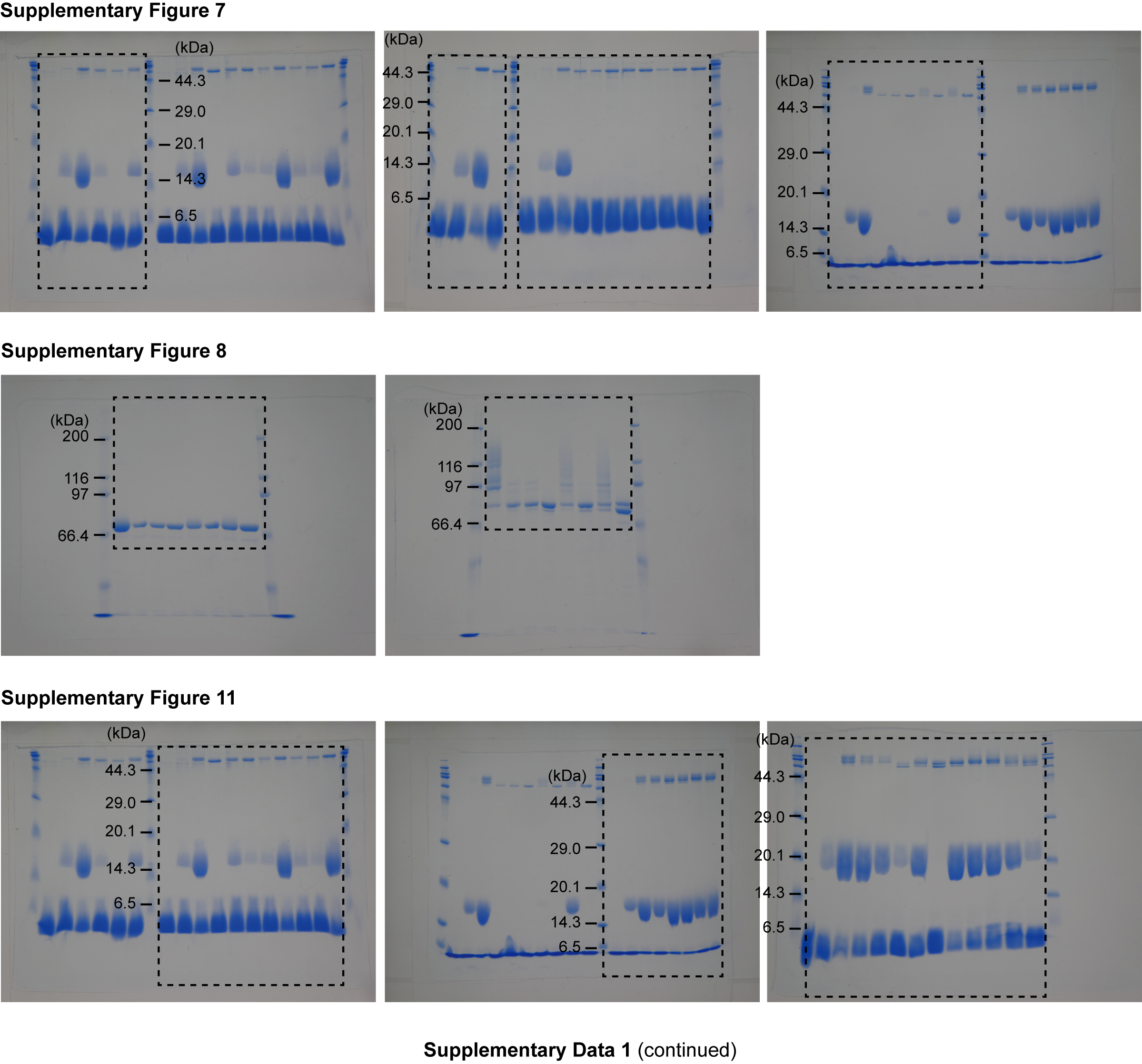


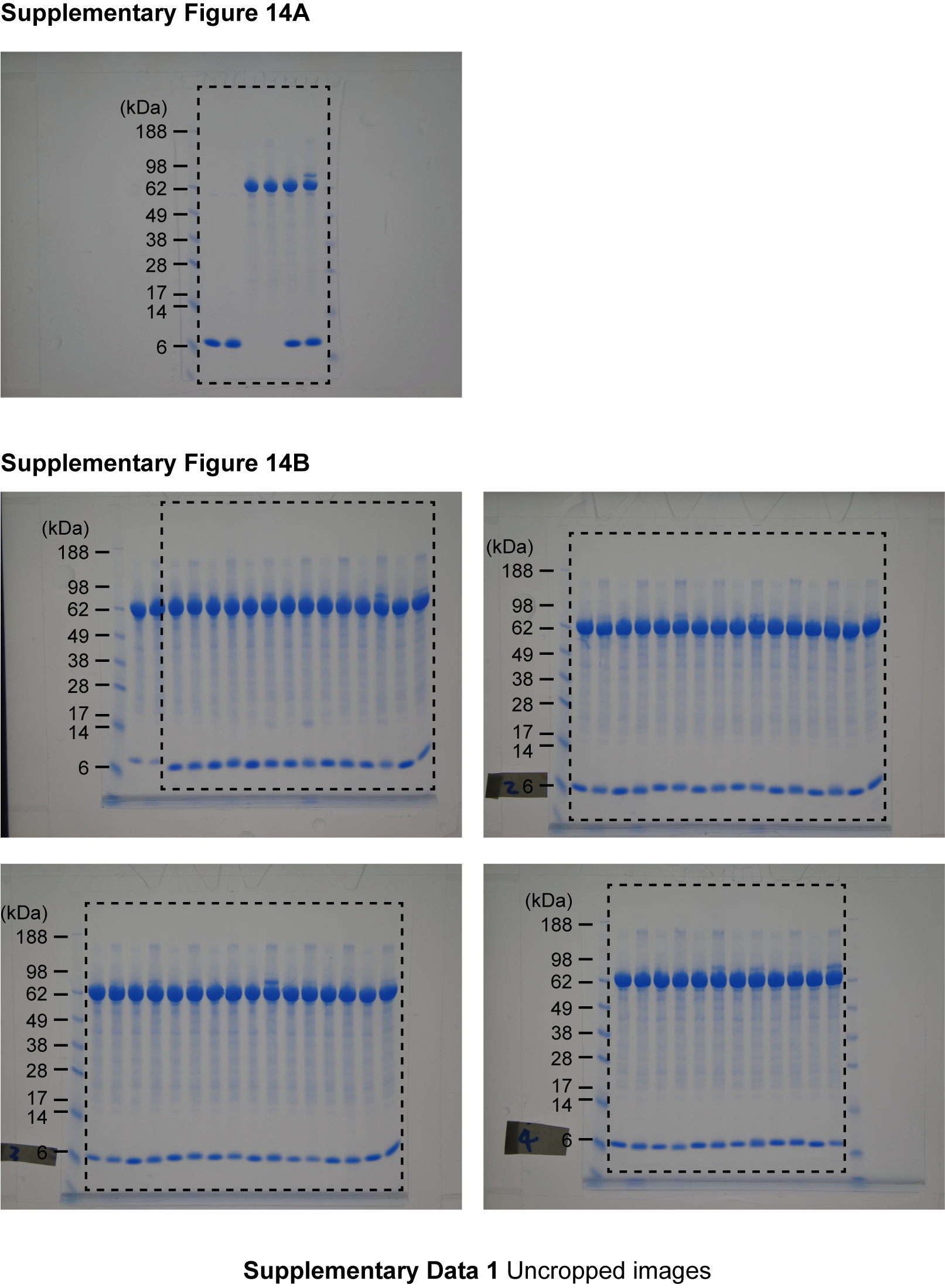

Supplement: Supplementary file 2 — Dataset 1 [file 41598_2018_28656_MOESM2_ESM.docx]
